# Tripartite motif 25 inhibits protein aggregate degradation during PRRSV infection by suppressing p62-mediated autophagy

**DOI:** 10.1128/jvi.01437-24

**Published:** 2024-10-31

**Authors:** Jiahui Ren, Qiming Pei, Haoxin Dong, Xuedan Wei, Liangliang Li, Hong Duan, Gaiping Zhang, Angke Zhang

**Affiliations:** 1 College of Veterinary Medicine, Henan Agricultural University, Zhengzhou, China; 2 International Joint Research Center of National Animal Immunology, College of Veterinary Medicine, Henan Agricultural University, Zhengzhou, China; 3 Ministry of Education Key Laboratory for Animal Pathogens and Biosafety, College of Veterinary Medicine, Henan Agricultural University, Zhengzhou, China; 4 College of Agronomy, Liaocheng University, Liaocheng, China; 5 Longhu Laboratory of Advanced Immunology, Zhengzhou, China; 6 Ministry of Education Key Laboratory for Animal Pathogens and Biosafety, Henan Agricultural University, Zhengzhou, China; University of Kentucky College of Medicine, Lexington, Kentucky, USA

**Keywords:** tripartite motif-containing 25, aggregates, autophagy, misfolded protein, porcine reproductive and respiratory syndrome virus

## Abstract

Viral infection causes endoplasmic reticulum stress and protein metabolism disorder, influencing protein aggregates formation or degradation that originate from misfolded proteins. The mechanism by which host proteins are involved in the above process remains largely unknown. The present study found that porcine reproductive and respiratory syndrome virus (PRRSV) infection promoted the degradation of intracellular ubiquitinated protein aggregates via activating autophagy. The host cell E3 ligase tripartite motif-containing (TRIM)25 promoted the recruitment and aggregation of polyubiquitinated proteins and impeded their degradation caused by PRRSV. TRIM25 interacted with ubiquitinated aggregates and was part of the aggregates complex. Next, the present study investigated the mechanisms by which TRIM25 inhibited the degradation of protein aggregates, and it was found that TRIM25 interacted with both Kelch-like ECH-associated protein 1 (KEAP1) and nuclear factor E2-related factor 2 (Nrf2), facilitated the nuclear translocation of Nrf2 by targeting KEAP1 for K48-linked ubiquitination and proteasome degradation, and activated Nrf2-mediated p62 expression. Further studies indicated that TRIM25 interacted with p62 and promoted its K63-linked ubiquitination via its E3 ligase activity and thus caused impairment of its oligomerization, aggregation, and recruitment for the autophagic protein LC3, leading to the suppression of autophagy activation. Besides, TRIM25 also suppressed the p62-mediated recruitment of ubiquitinated aggregates. Activation of autophagy decreased the accumulation of protein aggregates caused by TRIM25 overexpression, and inhibition of autophagy decreased the degradation of protein aggregates caused by TRIM25 knockdown. The current results also showed that TRIM25 inhibited PRRSV replication by inhibiting the KEAP1-Nrf2-p62 axis-mediated autophagy. Taken together, the present findings showed that the PRRSV replication restriction factor TRIM25 inhibited the degradation of ubiquitinated protein aggregates during viral infection by suppressing p62-mediated autophagy.

**IMPORTANCE:**

Sequestration of protein aggregates and their subsequent degradation prevents proteostasis imbalance and cytotoxicity. The mechanisms controlling the turnover of protein aggregates during viral infection are mostly unknown. The present study found that porcine reproductive and respiratory syndrome virus (PRRSV) infection promoted the autophagic degradation of ubiquitinated protein aggregates, whereas tripartite motif-containing (TRIM)25 reversed this process. It was also found that TRIM25 promoted the expression of p62 by activating the Kelch-like ECH-associated protein 1 (KEAP1) and nuclear factor E2-related factor 2 (Nrf2) pathway and simultaneously prevented the oligomerization of p62 by promoting its K63-linked ubiquitination, thus suppressing its recruitment of the autophagic adaptor protein LC3 and ubiquitinated aggregates, leading to the inhibition of PRRSV-induced autophagy activation and the autophagic degradation of protein aggregates. The present study identified a new mechanism of protein aggregate turnover during viral infection and provided new insights for understanding the pathogenic mechanism of PRRSV.

## INTRODUCTION

Cells adapt to detrimental conditions by inducing a wide range of sophisticated stress-response mechanisms when facing noxious stimulus such as oxidative stress, proteotoxicity stress, and viral infection (1). Protein homeostasis, which is controlled by cellular pathways responsible for protein synthesis, folding, trafficking, and degradation, is disrupted, and this is one of the most notable responses, leading to the production of numerous misfolded proteins (2). If unresolved, these misfolded proteins can further aggregate and interrupt normal cellular functions. To maintain proper cellular homeostasis, efficient clearance of misfolded proteins and protein aggregates is crucial. In most cases, misfolded proteins are degraded by the ubiquitin-proteasome system (UPS) or the autophagy pathway (3, 4). However, the proteasome is generally used for selective degradation of short-lived and abnormal/misfolded proteins following labeling with Lys-48-linked polyubiquitin chains (5). When the UPS is impaired or compromised, some misfolded proteins tend to form small cytosolic aggregates that are selectively transported to the aggresome, which consists of ubiquitinated proteins, chaperones, and proteasome components, sharing numerous characteristics with pathogenic inclusions in age-related degenerative diseases and serving as substrates for macroautophagy (hereafter referred to as autophagy) (6–8). Autophagy is generally responsible for the turnover of long-lived proteins and the clearance of aggregation-prone proteins (7). Thus, inactivation of autophagy leads to accumulation of cytoplasmic protein aggregates, which are composed of degenerated proteins, leading to various diseases such as neurodegeneration (9).

Autophagy selectively removes ubiquitinated and aggregated proteins and largely depends on the recognition of ubiquitinated and aggregated proteins by receptors such as p62/SQSTM1 (hereafter referred to as p62) and NBR1 (10). The p62 protein usually coexists with polyubiquitinated protein aggregates. In certain neurodegenerative diseases such as Lewy bodies in Parkinson’s disease and Huntingtin aggregates in Huntington disease, p62 is observed in ubiquitinated protein aggregates (11–13). The p62 protein localizes to sites of autophagosome formation and can associate with both the autophagosome localizing protein LC3 and ubiquitinated proteins (14, 15), facilitating the formation of protein aggregates due to its capacity of self-polymerization (14, 16). Aggresomes are degraded by selective autophagy (aggrephagy), and ubiquitinated and aggregated proteins recruited in aggresomes are degraded by p62-dependent selective autophagy (10).

The Kelch-like ECH-associated protein 1 (KEAP1)-Nrf2 pathway is well established as the main signaling cascade regulating cellular redox balance (17–19). Under normal physiological conditions, KEAP1 binds to and sequesters Nrf2 in the cytoplasm, resulting in the latter ubiquitination and proteasome degradation (20). Under stress stimulation conditions, Nrf2 is dissociated from KEAP1, translocating to the nucleus and activating the expression of target genes such as p62 (21).

TRIM family contains ~75 different protein members, most of which possess E3 ubiquitin ligase activity. Their typical structural characteristics consist of a conserved N-terminal RING domain, a B-box, and a coiled-coil domain (22). Some TRIM proteins have autophagy regulatory receptors function and are involved in modulating cell selective autophagy process. For example, TRIM5 is a selective autophagy receptor and is able to suppress HIV-1 replication (23). TRIM21-mediated precision autophagy targets cytoplasmic interferon regulatory factor 3 to regulate innate immunity (24). Besides, TRIMs have been reported to be associated with stress response-induced aggresomes formation and degradation (25–27). For example, TRIM25 promotes endoplasmic reticulum (ER)-associated degradation of oxidative stress proteins through targeting Keap1-Nrf2 pathway and thus facilitating cell survival and growth of hepatocellular carcinoma (28). Previous research has shown that TRIM proteins may provide an effective platform for the assembly of autophagy-related factors and autophagic cargos, including autophagy initiation (ULK1), extension (ATG16L1), and completion (LC3B) factors and is involved in the formation and degradation of protein aggregates (25). Even so, the role of TRIM family proteins in the autophagic degradation of ubiquitinated protein aggregates is still largely unknown.

Porcine reproductive and respiratory syndrome virus (PRRSV) is classified into the family *Arteriviridae* in the order *Nidovirales* and is an enveloped RNA virus (29) that mainly causes reproductive failure of sows and respiratory diseases of young pigs, thus remaining a major threat to global pork production (30). PRRSV infection can cause cell ER stress response, further leading to the disorder of the protein translation and modification system through a series of pathways, producing a large number of misfolded proteins in host cells (31). In addition to PRRSV, some other animal viruses, including porcine epidemic diarrhea virus (PEDV) and pseudorabies virus (PRV), can also induce unfolded protein response (UPR), causing the accumulation of misfolded proteins (32, 33). Misfolded proteins are harmful to the normal physiological function of host cells in the majority of cases and must therefore be removed in an appropriate manner. Thus far, the regulatory mechanism of ubiquitinated protein aggregates during PRRSV infection remains unknown, and whether TRIMs play a certain role in this process needs further clarification.

In the present study, the impact of PRRSV infection on protein aggregates formation and the molecular mechanisms by which host cells regulate the turnover of protein aggregates were explored. The current results showed that PRRSV infection induced the degradation of ubiquitinated protein aggregates by activating autophagy, while the host E3 ligase TRIM25 inhibited the clearance of aggregates by blocking p62-mediated autophagy. The present findings imply a novel function of TRIM25 in regulating protein homeostasis during PRRSV infection.

## MATERIALS AND METHODS

### Cells, virus, and stable cell line production

African green monkey kidney cells (Marc-145 cells, the only PRRSV permissive cell line *in vitro*) and HEK293T cells were cultured in Dulbecco's modified Eagle's medium (DMEM) supplemented with 10% fetal bovine serum (FBS), 100 U/mL penicillin, and 10 µg/mL streptomycin sulfate and maintained at 37°C in a humidified 5% CO_2_ incubator.

One highly pathogenic PRRSV strain (namely, SD16; GeneBank accession no. JX087437.1) was used in the present study and was propagated and titrated in Marc-145 cells. Briefly, Marc-145 cells were cultured in T25 cell culture bottles. When the cells reached 80% confluence, the culture medium was discarded, and 0.1 multiplicity of infection of PRRSV was inoculated into the cells and incubated at 37°C for 2 h. After discarding the unbound virus and washing with phosphate buffered saline (PBS) (cat. no. P1020; Beijing Solarbio Science & Technology Co., Ltd.), the cells were cultured with DMEM containing 3% FBS. When ~80% of cells exhibited a cytopathic effect, cells were harvested, frozen, and thawed three times at −80°C. Next, the cell suspension was centrifuged at 4°C at 12,000 rpm for 10 min to remove cell debris. Virus supernatants were titrated on Marc-145 cells seeded in a 96-well plate. Virus supernatants were 10-fold serially diluted, and 100 µL was added to each well (*n* = 8 replicates). Six days after the infection, the 50% cell culture infection dose was calculated by the Reed-Muench method.

HEK293T cells were transfected with psPAX2 (1.8 µg), pMD2.G (1.0 µg), and pTRIP-TRIM25-Flag (1.3 µg) or pTRIP-Vector for 48 h to produce pseudotyped lentiviral vectors using X-tremeGENE HP DNA transfection reagent (cat. no. 6366236001; Roche Diagnostics). Marc-145 cells were inoculated with pseudotyped lentivirus and selected for 2 weeks with 1 mg/mL puromycin (cat. no. A1113802; Invitrogen; Thermo Fisher Scientific, Inc.). For the establishment of an Nrf2 stable knockdown cell line, psPAX2 (1.8 µg), pMD2.G (1.0 µg), and pLKO.1-short hairpin (sh)Nrf2 (1.3 µg) or pLKO.1-Vector were co-transfected into HEK293T cells to produce pseudotyped lentivirus. The establishment of a recombinant knockdown cell line was performed as previously described (34).

### Reagents and antibodies

The following reagents were employed: MG132, a ubiquitin-proteasome inhibitor and autophagy activator (cat. no. S1748; Beyotime Institute of Biotechnology); 3-MA, an autophagy inhibitor (cat. no. HY-19312; MedChemExpress); Z-VAD-FMK, a cell apoptosis inhibitor (cat. no. HY-16658; MedChemExpress); cycloheximide (CHX), a protein translation inhibitor (cat. no. HY-12320; MedChemExpress); PP242, an autophagy activator (cat. no. HY-10474; MedChemExpress); rapamycin, an autophagy activator (cat. no. HY-10219; MedChemExpress); and a protein Aggresome Detection Kit (cat. no. ab139486; Abcam).

The following antibodies were used: Anti-TRIM25 polyclonal antibody (cat. no. 12573–1-AP; Proteintech Group, Inc.); anti-ubiquitin antibody (cat. no. 10201–2-AP; Proteintech Group, Inc.); anti-Myc antibody (cat. no. AF0033; Beyotime Institute of Biotechnology); anti-HA antibody (cat. no. AF0039; Beyotime Institute of Biotechnology); anti-KEAP1 antibody (cat. no. 10503–2-AP; Proteintech Group, Inc.); anti-Nrf2 antibody (cat. no. 16396–1-AP; Proteintech Group, Inc.); anti-p62 antibody (cat. no. 18420–1-AP; Proteintech Group, Inc.); anti-LC3 antibody (cat. no. 14600–1-AP; Proteintech Group, Inc.); anti-K48-ubiquitin antibody (cat. no. 30339; Signalway Antibody LLC); anti-K63-ubiquitin antibody (cat. no. 49420; Signalway Antibody LLC); anti-α-tubulin antibody (cat. no. T6074; Sigma-Aldrich; Merck KGaA); anti-HSP70 (cat. no. AF1156; Beyotime Institute of Biotechnology); anti-ATG5 (cat. no. AG4456; Beyotime Institute of Biotechnology); anti-Beclin1 (cat. no. AF5123; Beyotime Institute of Biotechnology); anti-green fluorescent protein (GFP) antibody (cat. no. AF0159; Beyotime Institute of Biotechnology); mouse mAbs 6D10 directed against PRRSV N protein (1 µg/mL; produced in our laboratory); horseradish peroxidase-conjugated goat anti-mouse IgG antibody (cat. no. 115–035-003; Jackson ImmunoResearch Laboratories, Inc.); horseradish peroxidase-conjugated goat anti-rabbit IgG antibody (cat. no. 111–035-003; Jackson ImmunoResearch Laboratories, Inc.); Alexa Fluor 488-conjugated goat anti-rabbit IgG (H&L; cat. no. ab150077; Abcam); and Alexa Fluor 594-conjugated goat anti-mouse IgG (H&L; cat. no. ab150116; Abcam).

### Plasmids and small interfering RNAs

pTRIP-TRIM25-Flag, pLenti-shTRIM25, pLenti-shNrf2, pCMV-p62-Myc, and pTRIP-KEAP1-Myc were constructed in our laboratory. GFP-LC3B was purchased from Addgene (cat. no. #11546). pCDH-EF1a-mCherry-EGFP-LC3B was purchased from Addgene (cat. no. #170446). pTRIP-TRIM25-ΔTRIP-flag, -ΔSPRY-HA, -PS-flag and -BBOX-flag were constructed in our laboratory. pTRIP-KEAP1-ΔBTB-Myc, -ΔDGR-Myc, and -ΔIVR-Myc were constructed in our laboratory. pCMV-p62-Myc and its corresponding mutants pEGFP-N1-p62_1–385_, -p62_120–440_, -p62_120–290_, -p62_270–385_, -p62_270–370_, and -p62_300–370_ were all constructed in our laboratory. HA-Ubiquitin, HA-Ubiquitin-K48, and HA-Ubiquitin-K63 were kindly provided by Professor Bo Wan (Henan Agricultural University, Zhengzhou, Henan province, China). pEGFP-N1-Nrf2 and pmTagBFP2-N1-p62 were constructed in our laboratory. Specific small interfering RNA (siRNA) targeting Nrf2, p62, LC3, ATG5, and Beclin1 and the corresponding control siRNA were purchased from Shanghai GenePharma Co., Ltd.

### Confocal microscopy

The treated cell samples were fixed using 4% paraformaldehyde (cat. no. 158127; Sigma-Aldrich; Merck KGaA) and then permeabilized with 0.3% Triton X-100 (cat. no. P0096; Beyotime Institute of Biotechnology) for 3 min at room temperature (RT). After blocking with 3% bovine serum albumin at RT for 1 h and washing with PBS, the cell samples were incubated with the indicated primary antibody at a dilution ratio of 1:500 at 4°C overnight, followed by washing with PBS three times and incubation with the corresponding fluorescence-conjugated secondary antibody at a dilution ratio of 1:500 for 1 h at RT in the dark. Coverslips were then mounted on glass slides with 4',6-diamidino-2-phenylindole (DAPI) counterstain (cat. no. F6057; Sigma-Aldrich; Merck KGaA) and were imaged using a Zeiss LSM510 laser scanning inverted confocal microscope (Zeiss AG) with a Zeiss 63X/1.4 NA oil immersion objective (Zeiss AG), and typical images were presented of the evaluated samples.

Cell samples that had undergone specific treatment were analyzed using fluorescence confocal microscopy to assess the formation of protein aggregates. Cells containing dispersed ubiquitin were considered as cells without aggregates, while cells containing obvious ubiquitin particles were considered as cells with aggregates. To determine the percentage of protein aggregates-positive cells, 20 fields were observed and recorded for each cell sample. To determine the diameter of ubiquitin-positive aggregates per sample, 20 individual fields were visualized in each sample using a Zeiss LSM510 confocal microscope (Zeiss AG). The size of aggregates was measured using Zeiss LSM Image Browser (Zeiss AG).

### Immunoprecipitation and western blotting

For immunoprecipitation (IP) assays, the treated cells were lysed in NP-40 lysis buffer (cat. no. P0013F; Beyotime Institute of Biotechnology) supplemented with protease inhibitor phenylmethanesulfonyl fluoride (PMSF) (1 mM; cat. no. ST506; Beyotime Institute of Biotechnology) on ice for 30 min, followed by centrifuging at 12,000 rpm for 10 min at 4°C to remove cell debris. The supernatants were incubated with Protein G Megbeads (cat. no. L00277; GenScript) pre-coated with the corresponding primary antibody at a dilution ratio of 1:50 overnight at 4°C on a tube rotator. The beads were washed with ice-cold PBS three times, and the proteins were eluted from the beads by boiling for 5 min in 2× SDS loading buffer and then subjected to western blot analysis.

### Extraction and assessment of aggregates formation in cell samples

To extract soluble proteins, mock- or PRRSV-infected or drug-treated cells were lysed using a lysis buffer containing 50 mM Tris (pH 8.0), 2% Triton X-100, 150 mM NaCl, 1 mM EDTA, 10% glycerol, protease inhibitor cocktail, and 1 mM PMSF. After centrifugation, the supernatant was considered the soluble fraction. To extract the insoluble fraction, the remaining cell lysate precipitate was washed with PBS and then dissolved with 8 M urea on a rotator overnight, followed by centrifugation at 12,000 rpm for 10 min at 4°C; the supernatant was aspirated and used as the insoluble protein fraction. After undergoing 10%–15% SDS-PAGE, the proteins were transferred to a polyvinylidene fluoride (PVDF) membrane (cat. no. ISEQ00010; MilliporeSigma). The blots were probed with specific antibodies at a dilution ratio of 1:3,000, which were visualized using ECL reagents (Tanon Biotech, China). The intensities of the target bands obtained were quantified using BandScan software, and α-tubulin was used as a loading control to calculate the relative quantity of target bands.

### CHX chase assay

CHX chase experiments were performed to assess protein stability in specific cells. Cells were treated with CHX (100 µg/mL) and harvested at 0, 2, 4, 6, 8, and 10 h post-CHX treatment. Cells were then harvested, lysed, and subjected to western blotting. The indicated protein levels were quantified with BandScan software to reflect protein stability.

### Virus titration

Virus progeny production was determined by titration. Briefly, Marc-145 cells were trypsinized and seeded in 96-well plate 24 h before virus infection. Virus supernatants were 10-fold serially diluted and added 100 µL to each well in eight repeated. Six days after infection, the 50% tissue culture infection dose (TCID_50_) was calculated by the Reed–Muench method.

### Statistical analysis

Data were analyzed using GraphPad Prism 8 software (GraphPad; Dotmatics), and two-tailed unpaired Student’s *t*-test was used to analyze data between two groups. The data are presented as the mean ± SD. All experiments were performed at least three times. *P* < 0.05 was considered to indicate a statistically significant difference.

## RESULTS

### PRRSV infection promotes aggregates degradation by activating autophagy

**Fig 1:**
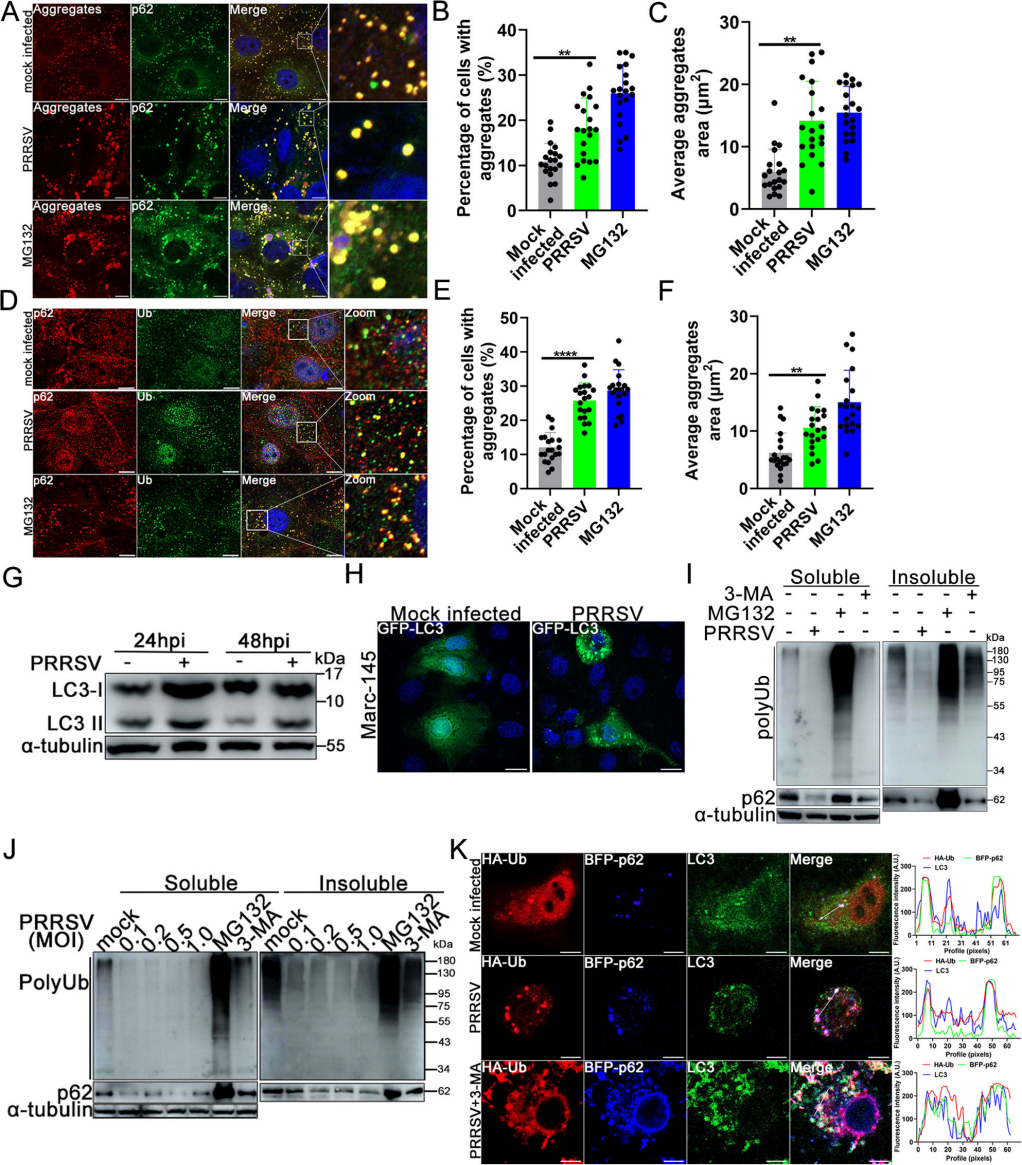
PRRSV infection promotes ubiquitinated protein aggregates degradation in host cells. (**A–C**) Confocal analysis of the effect of PRRSV infection on aggregates formation. Marc-145 cells were mock-infected or infected with PRRSV (MOI = 0.1) or treated with MG132 (10 µM). At 36 hpi, cells were fixed, permeabilized, and followed by staining with rabbit anti-p62 polyclonal antibody (pAb) and Alexa 488-conjugated goat anti-rabbit IgG (H + L; green), and protein aggregates were detected using an Aggresome Detection Kit (red). Scale bar: 10 µm. (**D–F**) Effect of PRRSV infection on p62-Ub double positive aggregates formation. PRRSV mock-infected, infected (0.1 MOI), or MG132 treatment Marc-145 cells were fixed and permeabilized. Then cells were visualized using rabbit anti-Ub pAb and Alexa 488-conjugated goat anti-rabbit IgG (H + L; green), mouse anti-p62 pAb, and Alexa 594-conjugated goat anti-mouse IgG (H + L; red). Scale bar: 10 µm. (**G and H**) Effect of PRRSV infection on intracellular autophagy activity. (**I**) Effect of PRRSV infection on ubiquitinated protein aggregates formation. Marc-145 cells were infected or not with PRRSV (MOI = 0.1). At 36 hpi, cells were harvested and lysated. After centrifugation, the clear supernatants were used as soluble fraction. Cell debris was dissolved in 8 M urea and was used as insoluble fraction. (**J**) Effect of different doses of PRRSV infection on aggregates formation. (**K**) Effect of PRRSV infection on p62 recruitment of ubiquitinated aggregates and LC3. 3-MA treatment group was set as the control. Three independent experiments were performed, and the percentage or volume of aggregates in 20 independent fields of cells was counted and calculated each time, and typical data were shown. ^**^, *P* < 0.01; ^***^, *P* < 0.001.

PRRSV infection triggers intracellular ER stress, leading to the formation of misfolded proteins (31). p62 Protein interacts with ubiquitinated misfolded proteins and promotes aggresomes formation (10). To determine the effect of PRRSV infection on intracellular protein aggregates, a protein Aggresome Detection Kit was employed. MG132, a proteasome inhibitor, was used as the positive control (35). In the mock-infected control group, p62-positive red fluorescent aggregates presented as a small particle-like distribution, while PRRSV infection prominently increased the proportion of cells with p62-positive protein aggregates (Fig. 1A and B) and simultaneously increased the volume of protein aggregates (Fig. 1C). As a positive control, MG132 treatment markedly increased the percentage of cells with p62-positive aggregates and the volume of p62-positive protein aggregates (Fig. 1A through C). Next, the status of ubiquitin (Ub)-p62 double-positive aggregates was investigated using an anti-ubiquitin antibody in the presence of PRRSV or not. Compared with the mock-infected cells, PRRSV infection substantially increased Ub-p62 double positive aggregates numbers and volumes (Fig. 1D through F). MG132 treatment also markedly increased Ub-p62 double positive aggregates numbers and volumes (Fig. 1D through F).

To determine the effect of PRRSV infection on aggregates content, the effect of PRRSV infection on autophagy was first analyzed. As shown in Fig. 1G and H, PRRSV infection markedly promoted autophagy activation of host cells, which was in accordance with previous research (36). Since PRRSV enhanced the formation of Ub-p62 double aggregates and activated autophagy, Marc-145 cells were infected with PRRSV, and the soluble and insoluble fractions of cell lysates were isolated and subjected to determination of intracellular ubiquitinated aggregates contents by western blotting. The results showed that, compared with the mock-infected control, PRRSV infection markedly decreased polyubiquitinated protein aggregates and p62 in the soluble fraction (Fig. 1I). In addition, there was a substantial decrease in polyubiquitinated protein aggregates and p62 in the insoluble fraction compared with the control group (Fig. 1I). MG132 treatment notably induced polyubiquitinated aggregates formation (Fig. 1I). 3-MA treatment also increased polyubiquitinated aggregates in the insoluble fraction (Fig. 1I). To further confirm the above results, Marc-145 cells were infected with different doses of PRRSV, and polyubiquitinated protein aggregates were analyzed. As shown in Fig. 1J, PRRSV infection dose-dependently prevented polyubiquitinated protein aggregates formation. Based on the above results, it was speculated that PRRSV infection promoted the recruitment of ubiquitinated protein aggregates to p62 (Fig. 1A and D), thereby activating autophagy to promote the degradation of these p62-positive aggregates. To corroborate such speculation, a confocal assay was performed to evaluate the association of ubiquitinated aggregates, p62, and the autophagic effector protein LC3B. As indicated in Fig. 1K, PRRSV infection significantly enhanced the co-localization of ubiquitinated aggregates, p62, and LC3B compared with control cells, while 3-MA treatment further enhanced their association, suggesting that PRRSV infection enhanced p62-mediated recruitment of ubiquitinated aggregates and LC3B and catalyzed the degradation of ubiquitinated aggregates by activating autophagy.

PRRSV infection induced the degradation of ubiquitinated aggregates, whether this phenomenon was in common between different domestic animal viruses, such as a typical DNA virus-PRV, was further determined. PRV infection caused accumulation of ubiquitinated aggregates in the insoluble fraction of porcine kidney (PK)−15 cells (Fig. S1), which was exactly opposite to PRRSV. As a positive control, MG-132 treatment induced aggregates accumulation, and 3-MA treatment also led to the increment of aggregates (Fig. S1). These results indicate that different viruses may induce degradation or accumulation of intracellular ubiquitinated aggregates.

### TRIM25 inhibits PRRSV-induced ubiquitinated aggregates degradation

**Fig 2:**
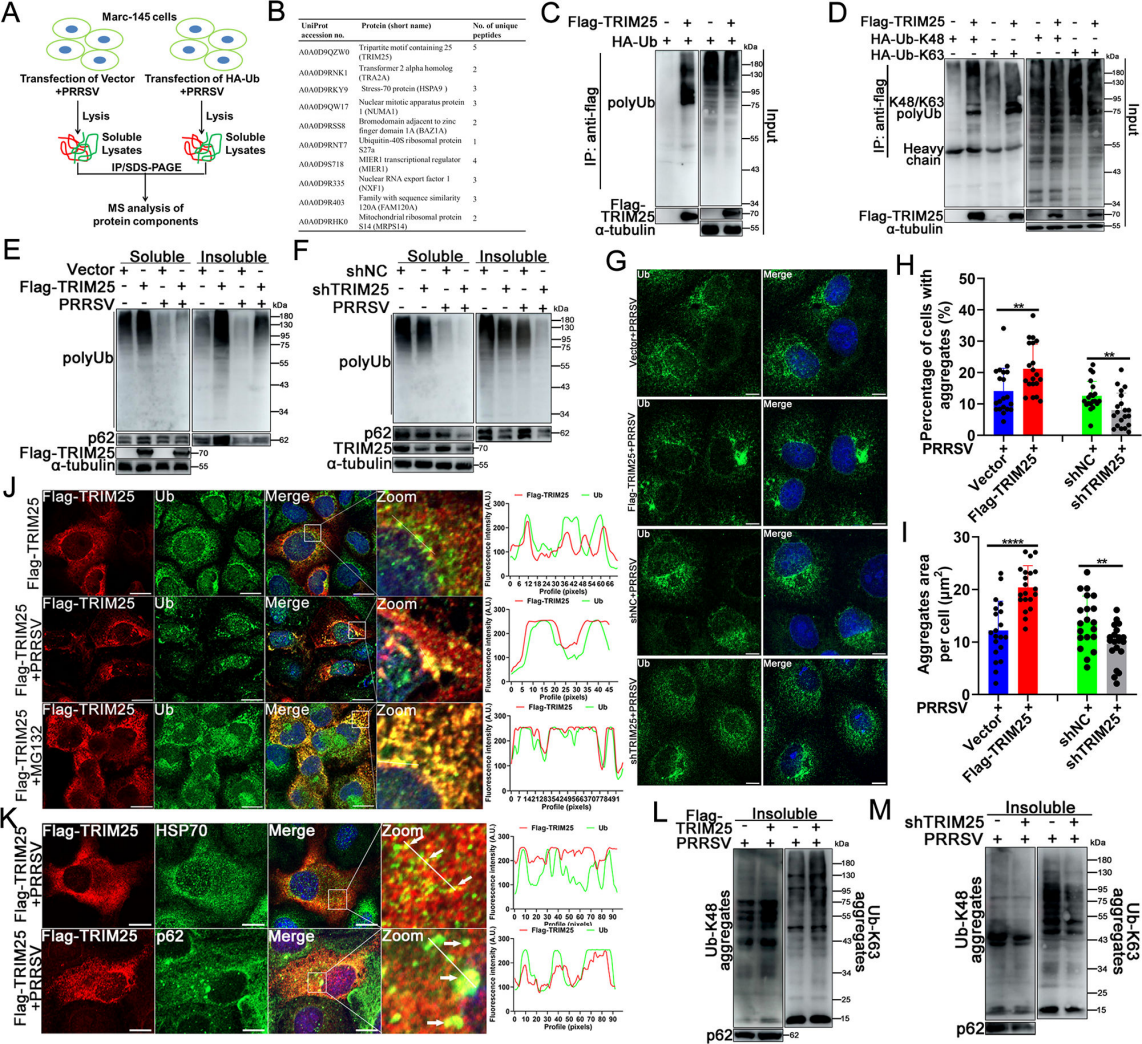
TRIM25 negatively regulated ubiquitinated aggregates degradation during PRRSV infection. (**A**) Scheme showing the procedure of identifying ubiquitination-related targets in response to PRRSV infection. (**B**) Marc-145 cells were transfected with or without HA-Ubiquitin for 12 h, and the supernatants were isolated and followed by IP using anti-HA antibody. Silver staining was performed, and the bands in corresponding Coomassie blue staining gel were analyzed by nano LC-MS/MS. (**C and D**) Analysis of interaction between TRIM25 and ubiquitinated protein. HA-Ub, -K48-Ub, and -K63-Ub plasmids were co-transfected into HEK293T cells or not with Flag-TRIM25 plasmids. At 48 h post-transfection (hpt), cells were collected, and co-IP was conducted for analysis of TRIM25 interaction with ubiquitinated protein aggregates. (**E and F**) Effect of TRIM25 overexpression (**E**) or knockdown (**F**) on aggregates clearance in the context of PRRSV infection. Vector, Flag-TRIM25, shNC, and shTRIM25 cells were mock-infected or infected with PRRSV (0.1 MOI). At 36 hpi, cells were harvested, and the soluble or insoluble fractions were isolated and subjected to western blotting analysis. (**G**) Confocal analysis of ubiquitinated aggregates. Vector, Flag-TRIM25, shNC, and shTRIM25 cells were infected with PRRSV at an MOI of 0.1. At 36 hpi, cells were fixed, permeabilized, and analyzed using rabbit anti-Ub pAb and corresponding fluorescent antibody. Scale bars: 10 µm. (**H and I**) Protein aggregates numbers and area analysis of average of 20 fields of Fig. 2G. Three independent experiments were performed, and typical data were shown. ^*^, *P* < 0.05; ^**^, *P* < 0.01; ^***^, *P* < 0.001. Scale bars: 10 µm. (**J**) Impact of PRRSV infection on the association of TRIM25 with protein aggregates. Flag-TRIM25 cells were left untreated, infected with a 0.1 MOI of PRRSV, or treated with 10 µM MG132. After infection or treatment for 24 h, cells were fixed, permeabilized, and detected using anti-flag monoclonal antibody (mAb), anti-Ub pAb, and corresponding fluorescent secondary antibody. Scale bars: 10 µm. (**K**) Co-localization analysis of TRIM25 with aggresome marker proteins. Flag-TRIM25 cells infecting with PRRSV (0.1 MOI) for 36 h were fixed, permeabilized, and detected with anti-Flag mAb, anti-p62, -HSP70 pAb, and corresponding fluorescent antibody. Scale bars: 10 µm. (**L and M**) Effect of TRIM25 on K48- and K63-linked ubiquitinated protein aggregates formation. Vector, Flag-TRIM25, shNC, and shTRIM25 cells were infected with PRRSV at an MOI of 0.1 for 36 h. The insoluble fractions were isolated and then subjected to western blotting analysis with anti-K48-Ub or -K63-Ub pAb.

To explore the possible function of host protein(s) in regulating PRRSV-mediated promotion of protein aggregates degradation, proteomics assays were designed to identify the potential candidate member(s) interacting with ubiquitinated proteins by IP together with nano-liquid chromatography-tandem mass spectrometry (LC-MS/MS) using Marc-145 cells transfected with HA-Ub expression plasmids (Fig. 2A). Among the protein(s) that might interact specifically with ubiquitinated protein aggregates, the present study focused on TRIM25 (Fig. 2B). To confirm the results of the interactome, co-IP was performed to test the interaction between TRIM25 and ubiquitinated aggregates. The co-IP results showed that TRIM25 pulled down ubiquitinated proteins (Fig. 2C), and both K48- and K63-linked ubiquitinated proteins were pulled down by TRIM25 (Fig. 2D), indicating that TRIM25 interacted with ubiquitinated protein aggregates.

Next, the present study assessed whether TRIM25 was able to regulate PRRSV-induced protein aggregates degradation using Marc-145-Vector, -TRIM25-flag, -sh negative control (NC), and -shTRIM25 cell lines (referred to as Vector, Flag-TRIM25, shNC, and shTRIM25, respectively; Fig. S2). Flag-TRIM25 or Vector control cells were infected with PRRSV, and the cellular soluble and insoluble fractions were extracted. Western blotting suggested that, although PRRSV infection decreased the level of ubiquitinated aggregates overall, overexpression of TRIM25 promoted the accumulation of ubiquitinated protein aggregates and p62 protein in the insoluble fraction compared with the Vector control group in the absence or presence of PRRSV (Fig. 2E).

Next, the effect of TRIM25 knockdown on PRRSV-induced aggregates degradation was evaluated. Knockdown of TRIM25 decreased ubiquitinated aggregates and p62 protein content in the insoluble fraction of cells with or without PRRSV infection (Fig. 2F). Confocal analysis showed that overexpression of TRIM25 increased ubiquitinated protein aggregates-positive cell percentage and aggregates volume during PRRSV infection (Fig. 2G through I), whereas knockdown of TRIM25 exerted the opposite effect (Fig. 2G through I). In addition, PRRSV infection enhanced the association of TRIM25 with ubiquitinated aggregates, as they formed larger yellow aggregates particles compared with mock-infected cells (Fig. 2J). As a positive control, MG132 treatment enhanced TRIM25 co-localization with aggregates and increased the volume of aggregates (Fig. 2J). The present results suggested that TRIM25 also co-localized with some aggresomal marker proteins such as HSP70 and p62 (Fig. 2K), further confirming that TRIM25 was closely associated with polyubiquitinated aggregates.

Next, the current study analyzed the types of ubiquitinated aggregates increased by TRIM25. Compared with the respective control cells, both K48- and K63-linked ubiquitinated protein aggregates increased in Flag-TRIM25 cells (Fig. 2L), while they decreased in shTRIM25 cells (Fig. 2M).

As TRIM25 promoted ubiquitinated aggregates accumulation, the effect of TRIM25 on endogenous ubiquitinated protein stability was evaluated. Flag-TRIM25 and shTRIM25 cell lines were both treated with the indicated doses of CHX, and the stability of ubiquitinated proteins was detected. The half-life of ubiquitinated proteins in Flag-TRIM25 cells was ~10 h (Fig. S3A and C), while it was 8 h in shTRIM25 cells (Fig. S3B and C), indicating that overexpression of TRIM25 markedly prolonged the half-life of ubiquitinated proteins. The E3 ligase activity of TRIM25 seemed to be indispensable for its association with aggregates since the TRIM25-ΔRING and -ΔSPRY mutants were both unable to co-localize with ubiquitin (Fig. S3D). Collectively, these findings suggest that TRIM25 is important for suppression of PRRSV infection-induced ubiquitinated aggregates degradation.

### TRIM25 interacts with p62 and promotes its expression via activation of the KEAP1-NRF2 pathway

Given that TRIM25 is an E3 ligase, conjoint analysis of transcriptomics and ubiquitinomics was performed to explore the underlying pathway responsible for the suppression of intracellular ubiquitinated aggregates degradation in response to PRRSV infection by TRIM25 (Fig. S4A). Kyoto Encyclopedia of Genes and Genomes analysis of the proteomics results suggested that the ubiquitin metabolic pathway was significantly regulated by TRIM25 overexpression, as the largest number of genes were enriched in this pathway (Fig. S4B). Consistently, gene ontology biological process enrichment analysis revealed that TRIM25 predominantly bound to molecules in the UPR pathway (Fig. S4C). These results identified the ubiquitin and UPR pathways as candidate signaling pathways regulated by TRIM25 during PRRSV infection.

Next, a combination of co-IP and MS was implemented to identify the potential target protein of TRIM25 associated with the regulation of the ubiquitin or UPR pathways and the biogenesis of protein aggregates. Of these possible target proteins, the present study focused on KEAP1 (Fig. S4D), which acts as a substrate-recognition subunit of the Cullin 3-based E3 ubiquitin ligase complex and an adapter protein targeting Nrf2 for ubiquitination and proteasome degradation (20, 37). The results showed that TRIM25 interacted with KEAP1 and promoted its proteasomal degradation via promoting the K48-linked ubiquitination of KEAP1 (Fig. S4E through S). As KEAP1 is a well-known inhibitor of Nrf2, and its interaction with Nrf2 functions as a key regulator of the oxidative stress pathway (18), the present study further explored the effect of TRIM25 on Nrf2 expression. The results showed that, during PRRSV infection, TRIM25 upregulated Nrf2 expression and activated the Nrf2 pathway through degrading KEAP1 and subsequent Nrf2 nuclear translocation (Fig. S5), which was consistent with previous reports (28). Considering that the co-IP combined with MS results indicated that TRIM25 pulled down another key autophagy-related protein, namely p62 (Fig. S4D), the present study next attempted to investigate whether TRIM25 may regulate the level of intracellular ubiquitinated aggregates via targeting the p62 pathway.

**Fig 3:**
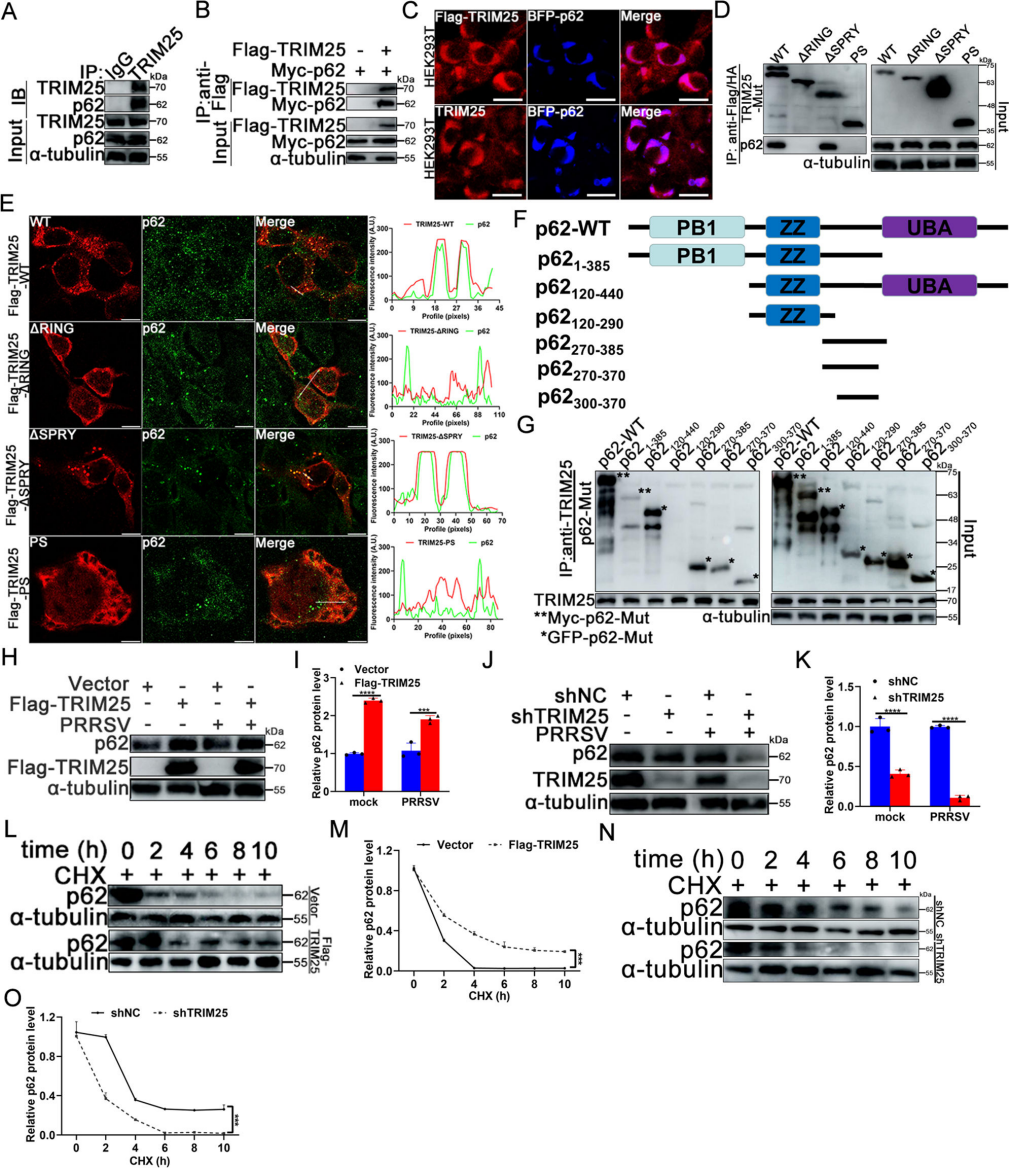
TRIM25 interacts with and promotes p62 expression. (**A**) The interaction between endogenous TRIM25 and p62 was assayed by co-IP. (**B**) Co-IP analysis of the interaction between exogenous TRIM25 and exogenous p62. (**C**) Confocal analysis of endogenous or exogenous TRIM25 co-localization with exogenous p62. Scale bar: 10 µm. (**D**) Mapping the domain(s) of TRIM25 interacting with full-length of p62. (**E**) Co-localization analysis of TRIM25 mutants with endogenous p62. Marc-145 cells were transfected with Flag-TRIM25-WT, -ΔRING, -PS, and HA-TRIM25-ΔSPRY, respectively, for 36 h. Cells were then fixed, permeabilized, and stained with anti-flag, -HA mAb, anti-p62 pAb, and corresponding fluorescent second antibody. Scale bar: 10 µm. (**F**) Constructs used for co-IP between full-length of TRIM25 and deletion mutants of p62. (**G**) Total lysates of HEK293T cells transfected with both p62 deletion mutant plasmids and Flag-TRIM25 plasmid were immuno-precipitated with anti-TRIM25 and immunoblotted with anti-Myc, -GFP, and anti-TRIM25 pAb. (**H–K**) Effect of TRIM25 overexpression or knockdown on p62 expression. (**L–O**) Vector, Flag-TRIM25, shNC, and shTRIM25 cells were treated with 100 mg/mL of CHX for 0, 2, 4, 6, 8, and 10 h, respectively, and cells were harvested for analysis of p62 expression.

To this end, the current study first determined whether TRIM25 interacted with p62. The co-IP results suggested that endogenous TRIM25 interacted with p62 (Fig. 3A). To further corroborate that TRIM25 was associated with p62, whether exogenous TRIM25 interacted with exogenous p62 was determined. The co-IP results showed that exogenous TRIM25 also interacted with exogenous p62 (Fig. 3B), and confocal microscopy showed similar subcellular localization of TRIM25 and p62 (Fig. 3C). Next, to map the domain(s) of TRIM25 involved in p62 interaction, a series of mutant plasmids of TRIM25 were co-transfected with Myc-p62 into HEK293T cells. It was found that the RING domain of TRIM25 was required for its efficient binding of p62 (Fig. 3D), and co-localization analysis of TRIM25 mutants with p62 exhibited similar results (Fig. 3E). To define the p62 interaction region, a series of p62 mutants were constructed, and co-IP assays were performed. As shown in Fig. 3F and G, the amino acid fragment 300–370 of p62 was identified as the key domain interacting with TRIM25, a domain that includes the LC3-binding region (38).

Next, the present study explored whether TRIM25 affected the expression of p62. The results showed that overexpression of TRIM25 enhanced p62 expression both in the absence and presence of PRRSV (Fig. 3H and I), while knockdown of TRIM25 decreased p62 expression (Fig. 3J and K). The current study next investigated whether TRIM25 affected the stability of p62 by using a CHX chase experiment. Overexpression of TRIM25 prolonged p62 half-life by enhancing its stability (Fig. 3L and M), whereas TRIM25 knockdown shortened p62 half-life by reducing its stability (Fig. 3N and O).

Since TRIM25 participated in the upregulation of p62, whether Nrf2 was associated with this process was investigated in the present study. Western blotting revealed that overexpression of Nrf2 promoted the expression of p62 (Fig. S6A), while knockdown of Nrf2 attenuated p62 protein level during PRRSV infection (Fig. S6B), suggesting that Nrf2 positively regulates p62 expression. As TRIM25 could activate the KEAP1-Nrf2 pathway (Fig. S5), these results suggested that TRIM25 upregulated the expression of p62 via activation of the KEAP1-Nrf2 pathway.

### TRIM25 suppresses p62 oligomerization

**Fig 4:**
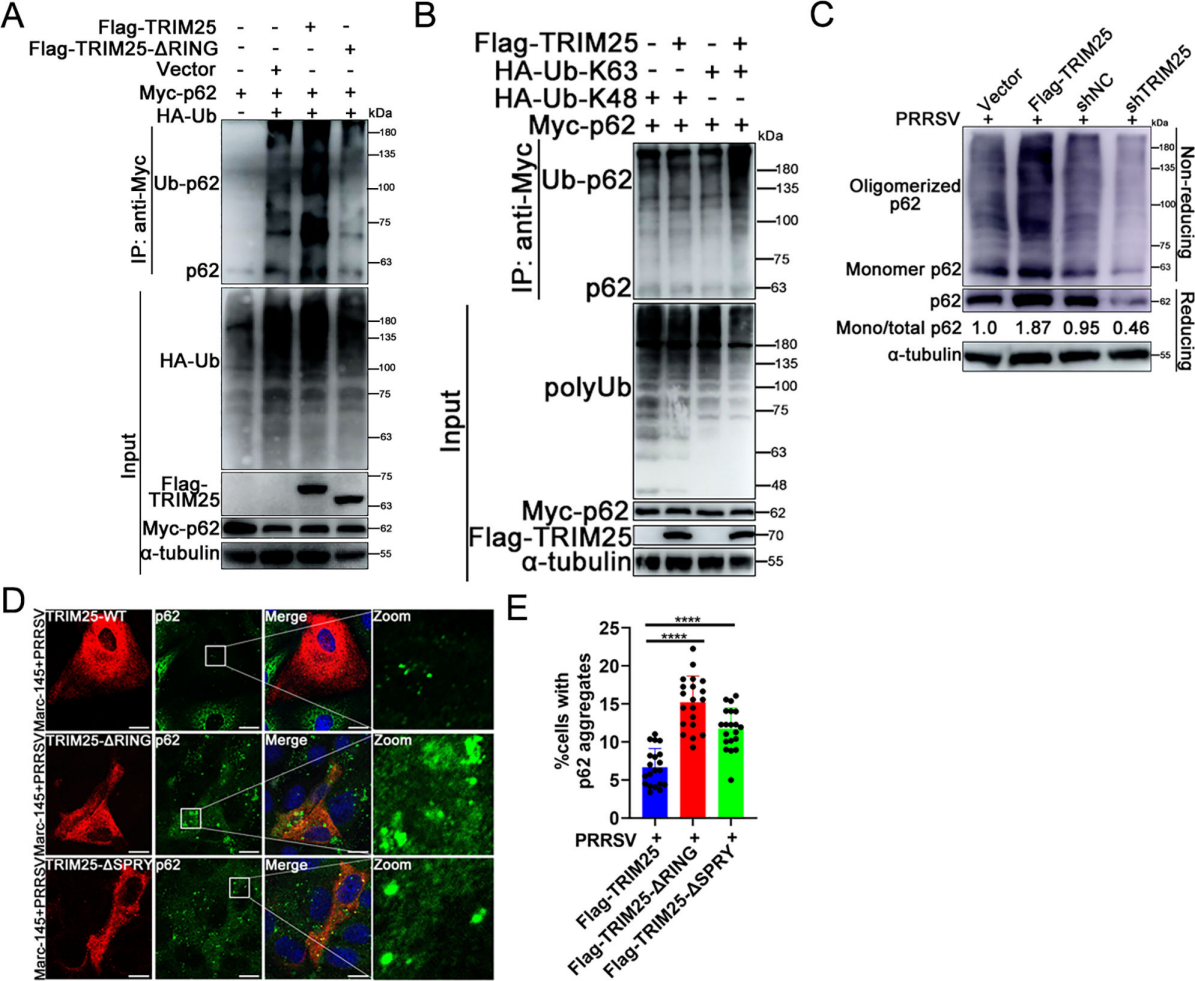
TRIM25 inhibits p62 oligomerization. (**A**) Myc-p62, HA-Ub, and Flag-TRIM25 or Flag-TRIM25-ΔRING plasmids were co-transfected into HEK293T cells, and the ubiquitination of p62 was analyzed by co-IP. (**B**) Co-IP analysis of K48- or K63-linked ubiquitination of p62 by TRIM25. (**C**) Vector, Flag-TRIM25, shNC, and shTRIM25 were cross-linked with 0.4 mg/mL dithiobis(succinimidylpropionate) at 4°C for 2 h, and the lysates were run under reducing or non-reducing conditions. (**D and E**) The key TRIM25 domain(s) affected the oligomerization of p62. Marc-145 cells were transfected with Flag-TRIM25-WT (500 ng/well), -ΔRING (500 ng/well), or -ΔSPRY (500 ng/well) mutant plasmids, respectively. At 12 hpt, the cells were infected with 0.1 MOI of PRRSV for 24 h. Cells were then fixed, permeabilized, and assayed for p62 oligomerization in transfection-positive cells. Scale bar: 10 µm. Data are mean ± SD values of three independent results. ^*^, *P* < 0.05; ^**^, *P* < 0.01; ^***^, *P* < 0.001; ns, not significant.

Previous studies have shown that ubiquitination modification of p62 affects its oligomerization, promoting its binding to polyubiquitinated proteins and sequestered them into aggregates for subsequent autophagic degradation (14, 39). To elucidate the molecular mechanism by which TRIM25 inhibiting PRRSV-induced aggregates degradation, the present study further investigated whether TRIM25 affected p62 protein ubiquitination and oligomerization in addition to its expression. The co-IP results showed that overexpression of TRIM25 significantly enhanced p62 ubiquitination, whereas the deletion of the RING domain of TRIM25 eliminated this effect (Fig. 4A). Further experiments revealed that TRIM25 had no marked influence on the K48-linked ubiquitination of p62, but it significantly enhanced its K63-linked ubiquitination (Fig. 4B). Previous research indicated that the K63-linked ubiquitination of p62 led to the inhibition of its dimerization, aggregation, and sequestration of ubiquitinated aggregates (40). Under reducing conditions, total p62 would be detected with a molecular weight of 62 kDa, whereas under non-reducing conditions, only p62 monomers would be detected at 62 kDa, while p62 oligomers or p62 conjugated with other proteins would display such a slow migration that it would not run through the gel. Therefore, a high ratio of p62 monomer protein in non-reducing vs reducing conditions would indicate reduced p62 oligomerization. Thus, the present study further explored the biological significance of the K63-linked ubiquitination of p62 by TRIM25. The results showed that overexpression of TRIM25 prevented p62 oligomerization, while knockdown of TRIM25 promoted p62 oligomerization (Fig. 4C). Confocal analysis of p62 oligomerization also revealed a similar phenomenon (Fig. S7). In the present study, TRIM25-wild-type (WT), -ΔRING, and -ΔSPRY were transfected into Marc-145 cells, and the domain(s) responsible for p62 oligomerization were determined. Confocal microscopy indicated that transfection with TRIM25-WT notably suppressed p62 oligomerization compared with surrounding normal cells (Fig. 4D, upper panel). By contrast, transfection of TRIM25-ΔRING or -ΔSPRY did not influence p62 oligomerization, while the degree of oligomerization of p62 was lower in TRIM25-ΔSPRY cells than in TRIM25-ΔRING cells (Fig. 4D and E), indicating that TRIM25 inhibited p62 oligomerization via its RING and SPRY domains.

### TRIM25 inhibits p62-mediated recruitment of the autophagic effector protein LC3

**Fig 5:**
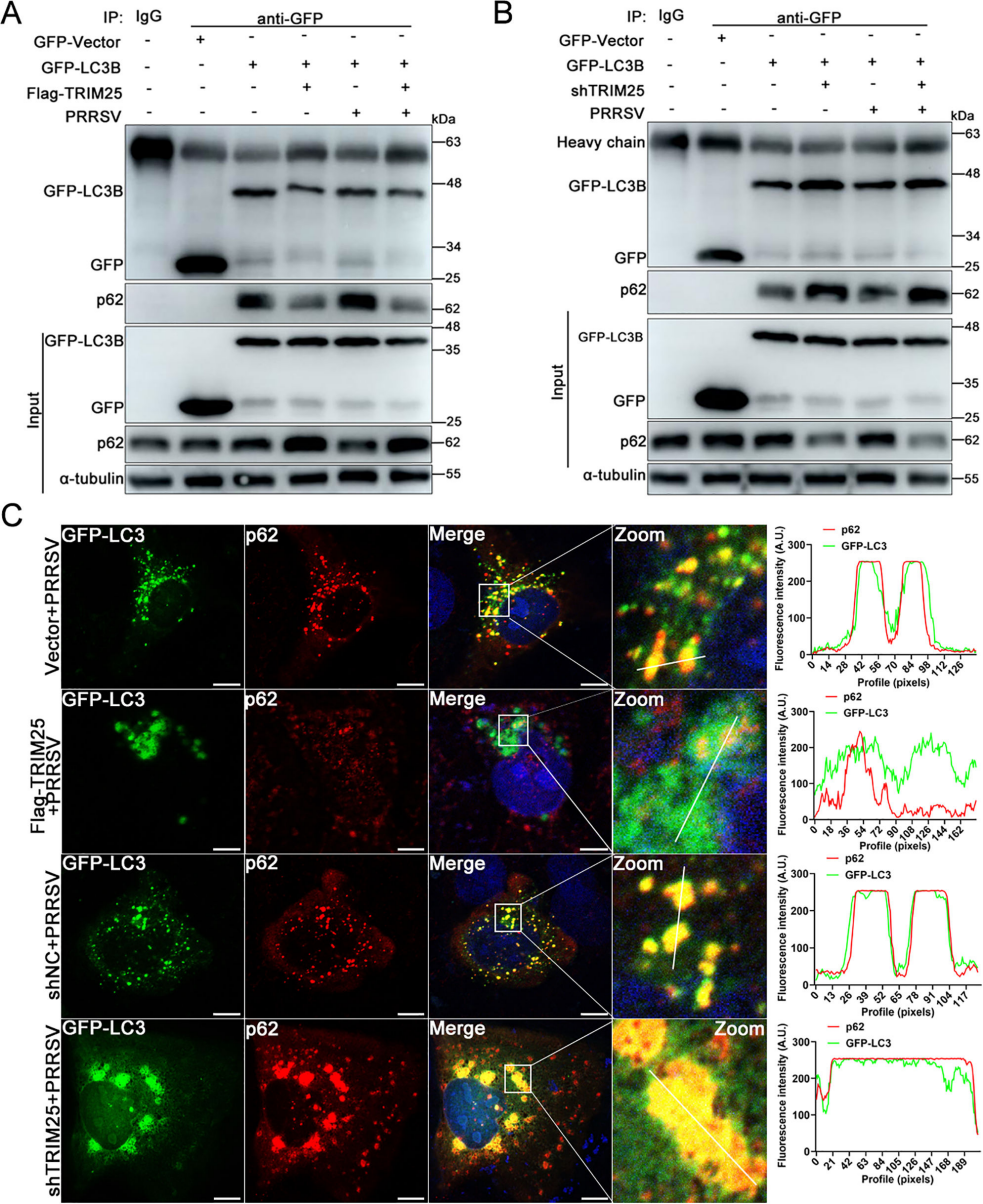
TRIM25 inhibits p62 recruitment of autophagic effector protein LC3. (**A**) Endogenous p62 co-immunoprecipitated with GFP-LC3B from transfected Vector and Flag-TRIM25 cell extracts infected or not with PRRSV (0.1 MOI). GFP or GFP-LC3B were immunoprecipitated from total cellular extracts of GFP-LC3B transfected (2 µg/well) Vector and Flag-TRIM25 cells and subjected to SDS-PAGE. Immunoprecipitated p62 or GFP-LC3B was detected by anti-p62 or -GFP antibody. (**B**) Endogenous p62 co-immunoprecipitated with GFP-LC3B from transfected shNC and shTRIM25 cell extracts infected or not with PRRSV (0.1 MOI). (**C**) Vector, Flag-TRIM25, shNC, and shTRIM25 cells were transfected with reporter plasmid GFP-LC3B (500 ng/well). At 12 hpt, cells were inoculated with 0.1 MOI of PRRSV and fixed at 36 hpi. p62 was stained using anti-p62 mAb and corresponding fluorescent antibody. Co-localization between p62 and GFP-LC3B was analyzed by confocal. Scale bar: 10 µm.

p62 Binding to LC3 is indispensable for the degradation of ubiquitinated protein aggregates by autophagy (38). Now that TRIM25 suppressed p62 oligomerization (Fig. 4), the present study further examined whether TRIM25 affected the binding affinity of de-oligomerized p62 to the autophagic effector protein LC3 using co-IP assay. Under normal physiological conditions, GFP-tagged LC3B pulled down endogenous p62 protein effectively, while overexpression of TRIM25 caused a decrease in the p62 level immunoprecipitated by LC3B (Fig. 5A). In addition, p62 immunoprecipitated by LC3B was also reduced by overexpression of TRIM25 during PRRSV infection (Fig. 5A). In the control sample, no clear target bands were pulled down by rabbit IgG (Fig. 5A). To further corroborate these results, shNC- and shTRIM25-transfected cells were used to determine the effect of TRIM25 knockdown on the p62-LC3B interaction. As expected, knockdown of endogenous TRIM25 both notably enhanced the p62 interaction with LC3B during PRRSV infection and in the absence of PRRSV infection (Fig. 5B). Whether TRIM25 affected p62 localization to autophagy-related structures was next analyzed by confocal microscopy using GFP-LC3B as a marker protein. As shown in Fig. 5C, partial p62 co-localized with LC3B during PRRSV infection, while overexpression of TRIM25 caused much less co-localization between p62 and LC3B. However, their co-localization was greatly enhanced when endogenous TRIM25 was knocked down (Fig. 5C, the bottom panel). Taken together, these results suggest that TRIM25 inhibited the p62-mediated recruitment of the autophagic effector protein LC3.

### TRIM25 suppresses autophagy activation

**Fig 6:**
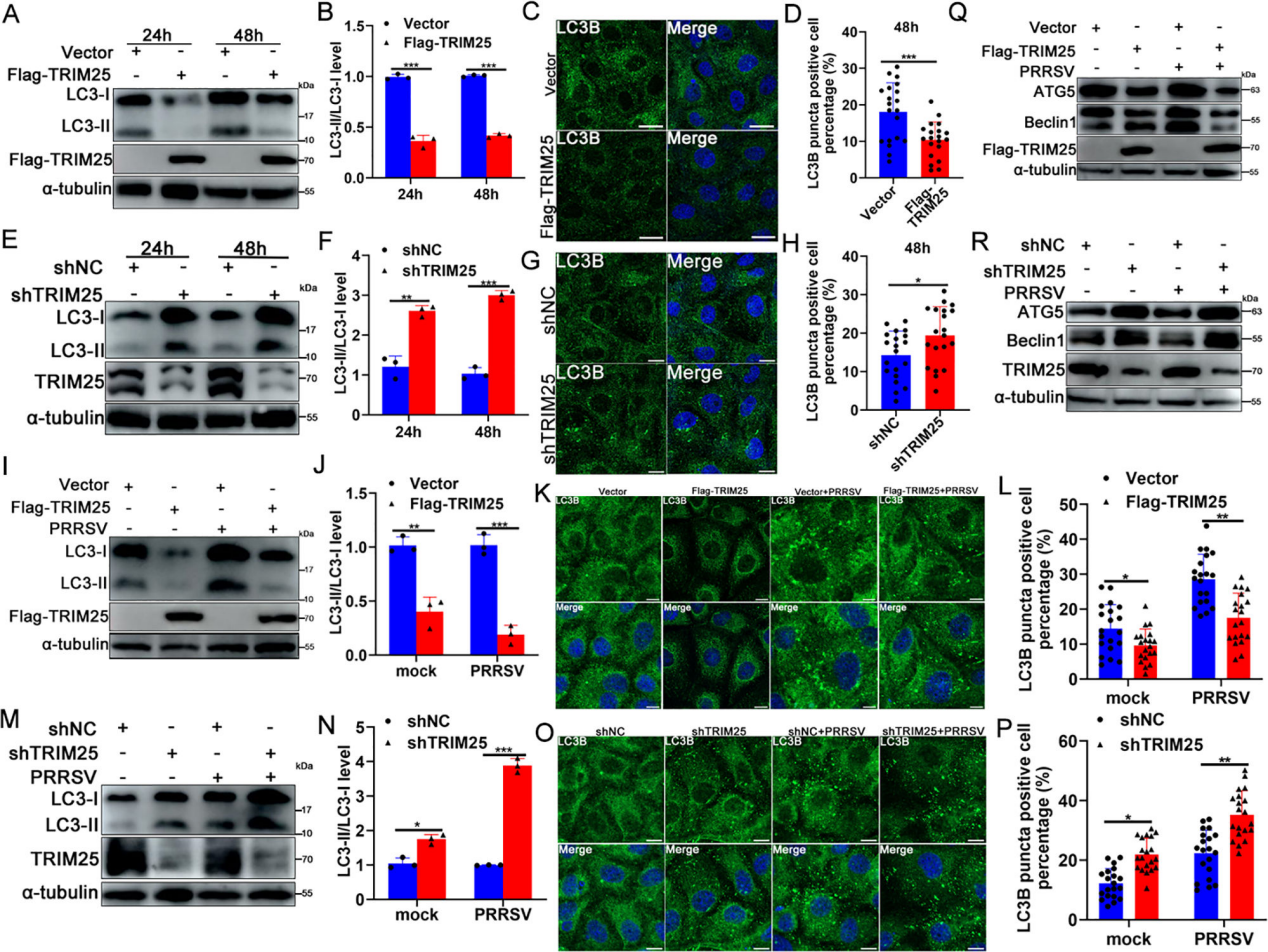
TRIM25 inhibits PRRSV-induced autophagy activation. (**A and B**) The protein levels of LC-I and LC3-II in Vector and Flag-TRIM25 cells at 24 and 48 h were assayed by western blotting. Data are mean ± SD values of three independent results. ^***^, *P* < 0.001. (**C and D**) Vector and Flag-TRIM25 cells were cultured for 48 h and then were fixed and assayed for the appearance of autophagosomes by staining with anti-LC3B mAb. ^***^, *P* < 0.001. Scale bars: 20 µm. (**E and F**) The protein levels of LC-I and LC3-II of shNC and shTRIM25 cells at 24 and 48 h were detected using western blotting. (**G and H**) Immunofluorescence stain of LC3B in shNC and shTRIM25 cells (**G**), and LC3B puncta positive cell percentage was analyzed of 20 independent fields, scale bar: 10 µm (**H**). (**I and J**) Vector and Flag-TRIM25 cells were inoculated or not with 0.1 MOI of PRRSV for 36 h. Cells were collected to determine the expression level of LC3-I and LC3-II. (**K and L**) Vector and Flag-TRIM25 cells were mock-infected or infected with 0.1 MOI of PRRSV for 36 h. LC3B puncta were stained using anti-LC3B mAb. (**M and N**) shNC and shTRIM25 cells mock-infected or infected with PRRSV (0.1 MOI) were analyzed for the expression of LC3-I and LC3-II. (**O and P**) LC3B puncta in PRRSV mock-infected or infected cells were analyzed using confocal. Scale bar: 10 µm. (**Q and R**) Vector, Flag-TRIM25, shNC, and shTRIM25 cells were inoculated with 0.1 MOI of PRRSV and cultured for 36 h. The expression of ATG5 and Beclin1 was analyzed by western blotting. For statistical analysis of relevant fluorescence images, three independent experiments were performed, 20 independent fields of cells were calculated each time, and typical data were shown. Data are mean ± SD values of three independent results. ^*^, *P* < 0.05; ^**^, *P* < 0.01; ^***^, *P* < 0.001.

**Fig 7:**
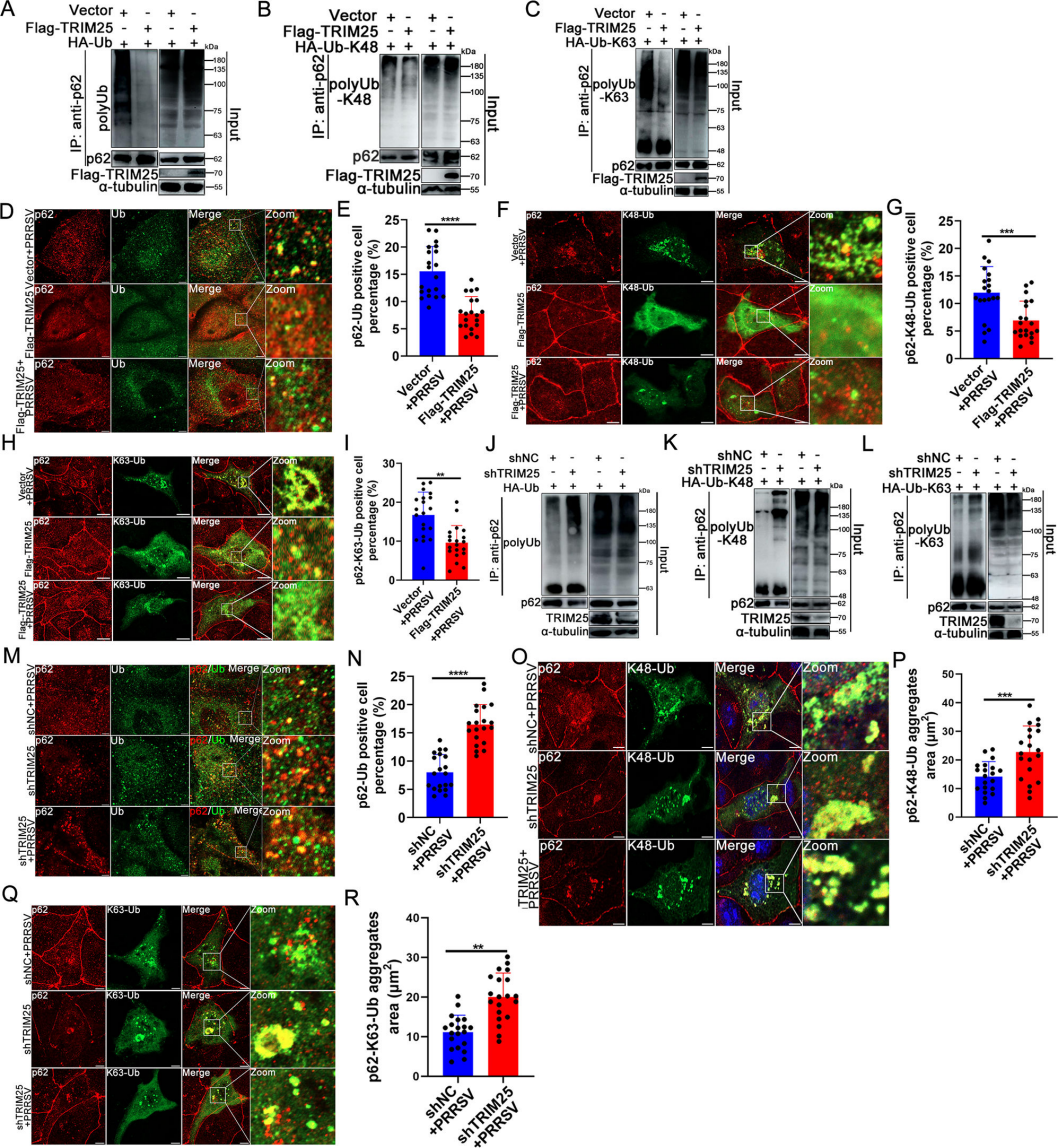
TRIM25 inhibits p62 recruitment of ubiquitinated protein aggregates. (**A–C**) Effect of TRIM25 overexpression on p62 binding to aggregates. IP of endogenous ubiquitinated aggregates in Vector or Flag-TRIM25 cells with anti-p62 antibody, IP products of polyUb (**A**), poly-K48-Ub (**B**), and poly-K63-Ub (**C**) were detected with anti-HA and -p62 mAb. (**D, F, and H**) Immuno-staining of p62 and Ub-linked protein (**D**) and K48- and K63-Ub-linked protein (**F and H**) in Vector or Flag-TRIM25 cells mock-infected or infected with PRRSV (0.1 MOI). (**E, G, and I**) Quantification of p62-, p62-K48-, or p62-K63-Ub double positive foci corresponding to group D, F, and H. (**J–L**) Effect of TRIM25 knockdown on p62 binding to aggregates. IP of endogenous ubiquitinated aggregates using anti-p62 antibody in shNC and shTRIM25 cells infected with PRRSV. IP products of polyUb (**J**), poly-K48-Ub (**K**), and poly-K63-Ub (**L**) were detected using specific antibody against HA or p62. (M, O, and Q) Confocal analysis of endogenous p62 recruiting Ub-linked protein (**M**) and K48- and K63-Ub-linked ubiquitinated protein (**O and Q**). (**N, P, and R**) Quantification of p62-, p62-K48-, or p62-K63-Ub double positive foci corresponding to group M, O, and Q. For statistical analysis of relevant fluorescence images, three independent experiments were performed, the p62-K48/-K63-Ub area in 20 independent fields of cells was calculated each time, and typical data were shown. ^**^, *P* < 0.05; ^***^, *P* < 0.01; ^****^, *P* < 0.001. Scale bar: 10 µm.

Since TRIM25 inhibited the p62-mediated recruitment of the autophagic protein LC3, the present study further investigated whether TRIM25 inhibited autophagy activity. To this end, the autophagic flux was evaluated first using Vector, Flag-TRIM25 or shNC, and shTRIM25 cell lines infected or not with PRRSV. Overexpression of TRIM25 decreased the level of LC3-II (which was derived from LC3-I) in the absence of PRRSV (Fig. 6A and B), while knockdown of TRIM25 increased LC3-II levels (Fig. 6E and F), indicating that TRIM25 inhibited constitutive autophagy activity. When these cell lines were infected with PRRSV, which can activate autophagy, the LC3-II level in Flag-TRIM25 cells was markedly lower than that in control cells (Fig. 6I and J), whereas it was markedly higher in shTRIM25 cells compared with control cells (Fig. 6M and N), suggesting a reduced autophagy flux caused by TRIM25. To confirm such findings, LC3B puncta numbers were analyzed by indirect immunofluorescence assay to further evaluate autophagy levels. The results revealed that overexpression of TRIM25 significantly decreased the LC3 puncta numbers (Fig. 6C and D), while knockdown of TRIM25 enhanced such numbers (Fig. 6G and H). In addition, overexpression of TRIM25 inhibited PRRSV-induced autophagy activation (Fig. 7K and L), while knockdown of TRIM25 enhanced autophagy activation (Fig. 6O and P).

Various critical junction proteins of the autophagy pathway were also analyzed. As expected, in the absence or presence of PRRSV infection, overexpression of TRIM25 suppressed the expression of the autophagy marker proteins ATG5 and Beclin1 (Fig. 6Q), while knockdown of TRIM25 enhanced their expression (Fig. 6R), suggesting a suppression of the autophagy process. These results suggested that the upregulation of p62 by TRIM25 does not activate intracellular autophagy; by contrast, the autophagy process is suppressed.

To further explore the role of TRIM25 during autophagosome formation and related processes, Flag-TRIM25 or shTRIM25 cells were transfected with the mCherry-eGFP-LC3B reporter plasmid, which emits red fluorescence in acidic vesicles (autolysosomes) and emits yellow fluorescence in neutral structures (autophagosomes). Subsequently, cells were infected with PRRSV. The number of fluorescent dots in different cells was statistically analyzed. Compared with control cells, although the numbers of yellow fluorescence protein (YFP)-positive dots increased (13 vs 23), the number of red fluorescence protein (RFP)-positive dots significantly decreased in Flag-TRIM25 cells (19 vs 8) in ~20 fields (Fig. S8A and B). On the other hand, knockdown of endogenous TRIM25 markedly increased the number of RFP-positive dots (21 vs 28), while the number of YFP-positive dots did not markedly change (17 vs 19) compared with control cells (Fig. S8A and B). These results indicated that TRIM25 directly decreased autophagic processes in target cells, and its downregulation increased autophagy processes.

To investigate whether TRIM25-mediated suppression of autophagy was universal, Vector or Flag-TRIM25 cells were subjected to starvation or rapamycin treatment and followed by analysis of autophagy flux using western blotting. The results showed that Vector cells treatment with rapamycin activated autophagy, as revealed by increased LC3II/LC3I ratio (Fig. S9A); however, overexpression of TRIM25 notably decreased LC3II/LC3I ratio, indicating the suppression of autophagy. Similarly, starvation also activated autophagy, and overexpression of TRIM25 inhibited starvation-induced autophagy activation (Fig. S9B). These results indicate that the suppression of TRIM25 on autophagy is universal intracellular phenomenon.

### TRIM25 inhibits the p62-mediated recruitment of ubiquitinated protein aggregates

In the autophagy-mediated degradation pathway, the polyubiquitin chain is recognized by proteins such as p62 and receptor proteins that recruit substrates to autophagosomes for degradation (41). Previous research has revealed that oligomerized p62 exhibits high affinity to substrates (40), and the current results showed that TRIM25 inhibited p62 oligomerization (Fig. 4). Therefore, the current study investigated the capability of p62 binding to ubiquitinated proteins in Flag-TRIM25 and shTRIM25 cells. Co-IP was performed using beads coated with an anti-p62 specific antibody. Although the overexpression of TRIM25 significantly promoted ubiquitinated protein accumulation (Fig. 2), the amount of p62 immunoprecipitated ubiquitinated protein notably decreased in this cell line (Fig. 7A), and both of the levels of immunoprecipitated K48- and K63-linked ubiquitinated protein decreased (Fig. 7B and C). Consistent with this, the proportion of p62-Ub double-positive aggregates and p62-K48- or p62-K63-Ub double-positive protein aggregates in Flag-TRIM25 cells during PRRSV infection decreased compared with those of control cells (Fig. 7D through I). By contrast, knockdown of endogenous TRIM25 expression enhanced the capability of p62 recruiting ubiquitinated proteins (Fig. 7J), although it reduced the production of ubiquitinated proteins due to TRIM25 knockdown (Fig. 2F). Besides, both K48- and K63-linked ubiquitinated protein recruitment by p62 increased (Fig. 7K and L). Confocal analysis of the p62-Ub, p62-K48-, or p62-K63-Ub double positive aggregates also revealed that knockdown of TRIM25 augmented the p62-mediated recruitment of these two types of aggregates (Fig. 7M through R). Taken together, these results suggest that TRIM25 inhibits the p62-mediated recruitment of ubiquitinated protein aggregates.

### TRIM25 blocks aggregates degradation by inhibiting autophagy

**Fig 8:**
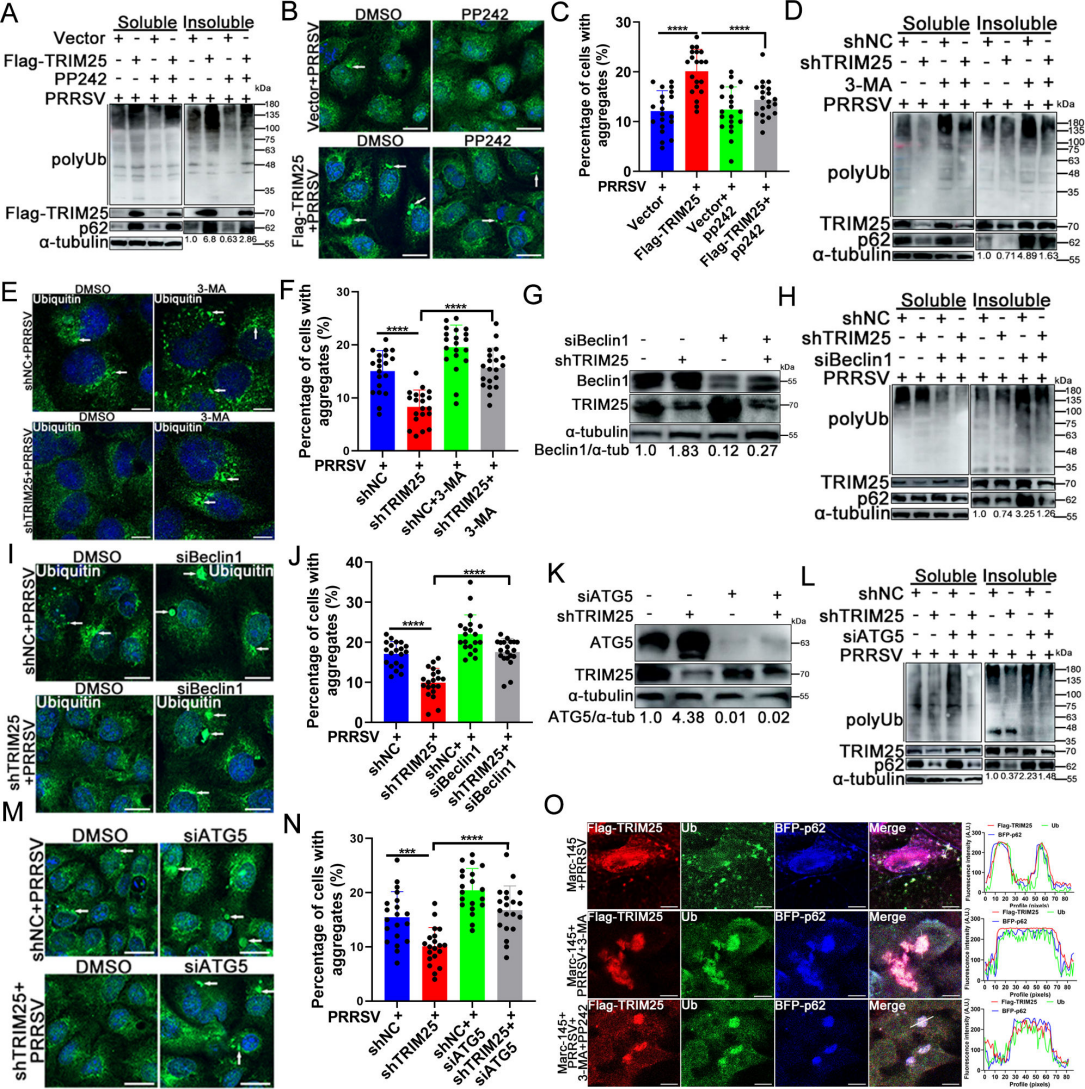
Inhibition of autophagy by TRIM25 promotes ubiquitinated aggregates accumulation. (**A–C**) Vector and Flag-TRIM25 cellls were infected with 0.1 MOI of PRRSV. At 24 hpi, cells were treated with dimethyl sulfoxide (DMSO) or 10 nM of PP242 for another 12 h. Soluble and insoluble fractions of cells were extracted and analyzed using western blotting. Part of the cell samples were fixed and assayed using confocal. ^***^, *P* < 0.001. Scale bar: 10 µm. (**D–F**) Effect of autophagy inhibition on ubiquitinated aggregates clearance. shNC and shTRIM25 cells infected with 0.1 MOI of PRRSV were treated with 10 mM of 3-MA for 12 h, and then aggregates were analyzed using western blotting and confocal, respectively. ^***^, *P* < 0.001. Scale bar: 10 µm. (**H–J**) Effect of Beclin1 knockdown on clearance of ubiquitinated aggregates. ^***^, *P* < 0.001. Scale bar: 10 µm. (**L–N**) Effect of ATG5 knockdown on clearance of ubiquitinated aggregates. ^***^, *P* < 0.001. Scale bar: 10 µm. (**G and K**) The protein level of Beclin1 and ATG5 was both assayed by western blotting after transfection with specific siRNA. (**O**) Confocal images of PRRSV-infected Marc-145 cells transfected with Flag-TRIM25, BFP-p62, and untreated or treated with PP242 (10 nM, 12 h) and/or 3-MA (10 mM, 12 h), and confocal was performed with antibodies as indicated. Right panel graphs: fluorescence intensity line is tracing corresponding to a white line in zoom panel. Scale bar: 10 µm. For statistical analysis of relevant fluorescence images, three independent experiments were performed, 20 independent fields of cells were calculated each time, and typical data were shown.

TRIM25 inhibited p62 oligomerization (Fig. 4) to prevent its recruitment of LC3 (Fig. 5) and ubiquitinated protein aggregates (Fig. 7) and suppressed the autophagic activity (Fig. 6). To further determine whether the autophagy inhibition caused by TRIM25 was responsible for the accumulation of ubiquitinated protein aggregates during PRRSV infection, Flag-TRIM25 cells infected with PRRSV were treated or not with the autophagy activator PP242, and ubiquitinated protein aggregates were analyzed. Western blot detection of ubiquitinated aggregates in the insoluble cell fraction showed that PP242 treatment largely decreased the level of aggregates in the insoluble cell fraction, although more aggregates were observed in Flag-TRIM25 cells than in control cells (Fig. 8A). Confocal microscopy results showed that the percentage of cells containing ubiquitinated protein aggregates was higher in Flag-TRIM25 cells than in control cells (Fig. 8B and C). However, after treatment with PP242, the percentage of aggregate-positive cells decreased in both cell lines, and the levels of aggregates in PP242-treated Flag-TRIM25 cells were much lower than in cells without PP242 treatment (Fig. 8B and C).

Next, shTRIM25 cells were treated with the autophagy inhibitor 3-MA to analyze the level of ubiquitinated protein aggregates. Western blot analysis of ubiquitinated protein aggregates in the insoluble fraction showed that 3-MA treatment prevented TRIM25 knockdown-induced aggregates clearance (Fig. 8D). Knockdown of TRIM25 reduced protein aggregates; however, aggregates were retained in numerous shNC and shTRIM25 cells treated with 3-MA, and the percentage of aggregates-positive cells was much higher in shTRIM25 cells treated with 3-MA than in cells without 3-MA treatment (Fig. 8E and F).

The expression of endogenous ATG5 or Beclin1, which are essential proteins in the autophagy process, was also knocked down in shNC and shTRIM25 cells using specific siRNAs. Western blot analysis of ATG5 and Beclin1 expression suggested that both two proteins were effectively inhibited by siRNA (Fig. 8G and K). Western blot analysis of protein aggregates in the insoluble cell fraction revealed that knockdown of Beclin1 inhibited ubiquitinated protein aggregates clearance in shTRIM25 cells since the levels of aggregates in shTRIM25 cells transfected with siBeclin1 were higher than in cells transfected with siNC (Fig. 8H). Confocal microscopy also showed that knockdown of Beclin1 blocked aggregates clearance in shTRIM25 cells (Fig. 8I and J). Knockdown of ATG5 inhibited ubiquitinated protein aggregates clearance in the insoluble fraction of shTRIM25 cells (Fig. 8L). Confocal microscopy also showed similar results (Fig. 8M and N). In addition, high levels of TRIM25 were observed in the insoluble fraction (Fig. 8A, D, H and L), suggesting that TRIM25 was present in the ubiquitinated aggregates. To further confirm that TRIM25 was physically present in the aggregates and participated directly in their turnover, the association between TRIM25, ubiquitin, and p62 was determined by confocal microscopy. Under basal conditions, TRIM25 co-localized with ubiquitinated aggregates and p62 (Fig. 8O, upper panel). After 3-MA treatment, the co-localization between TRIM25, aggregates, and p62 was markedly enhanced (Fig. 8O, middle panel), while treatment with PP242 for 12 h followed by 3-MA treatment for additional 12 h significantly reduced their co-localization, particularly that of aggregates and p62 fluorescence signals, while having no significant effect on the TRIM25 signal (Fig. 8O, lower panel), further demonstrating that TRIM25 inhibited aggregates degradation during PRRSV infection by inhibiting autophagy. Taken together, these results support the hypothesis that TRIM25-induced autophagy inhibition directly hinders the clearance of ubiquitinated protein aggregates during PRRSV infection.

### TRIM25 inhibits PRRSV replication via inhibiting the KEAP1/Nrf2/p62-mediated autophagy

**Fig 9:**
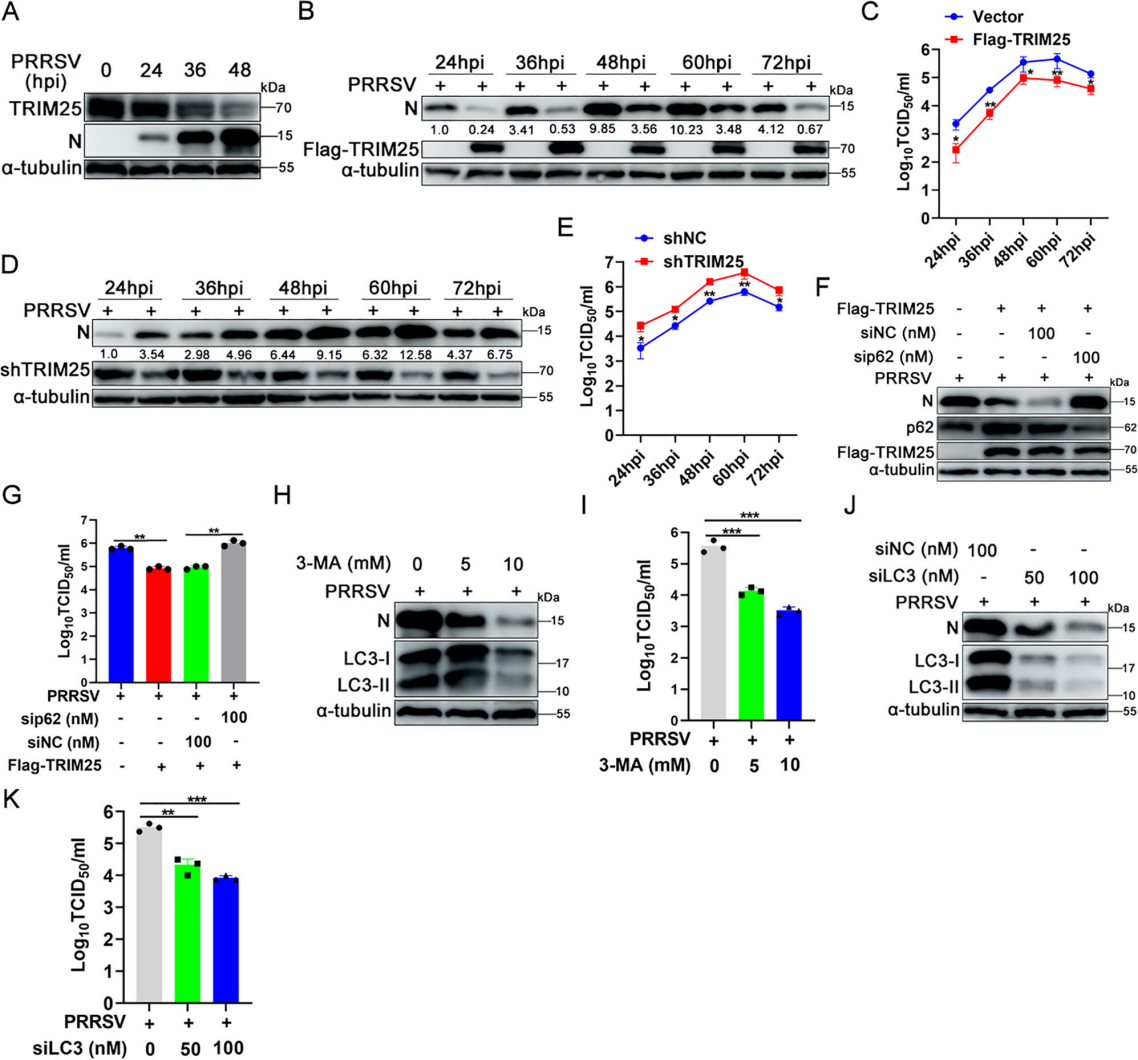
TRIM25 inhibits PRRSV replication. (**A**) Marc-145 cells infected with PRRSV or not were harvested at 24, 36, and 48 hpi, respectively, and then analyzed by western blotting. (**B and C**) Vector and Flag-TRIM25 cells were infected with 0.1 MOI of PRRSV. At 24, 36, 48, 60, and 72 hpi, cells and supernatants were collected to detect the expression of PRRSV N protein and progeny viral titers using western blotting and TCID_50_ assay, respectively. (**D and E**) shNC and shTRIM25 cells infected with 0.1 MOI of PRRSV were collected at 24, 36, 48, 60, and 72 hpi to analyze the level of PRRSV N protein and progeny viral titers using western blotting and TCID_50_ assay, respectively. (**F and G**) Flag-TRIM25 cells were transfected with 100 nM siNC or sip62 for 12 h and then infected with PRRSV (MOI = 0.1). Cells were collected at 36 hpi to detect the expression of RRSV N protein using western blotting; and for viral replication kinetics analysis, cell supernatants were collected at 36 hpi to detect progeny viral titers using TCID_50_ assay. (**H and I**) Marc-145 cells infected with 0.1 MOI of PRRSV were treated with DMSO, 5 and 10 mM of 3-MA from 1 hpi and onwards. At 36 hpi, the expression of PRRSV N protein was analyzed using western blotting; for viral replication kinetics analysis, supernatants were collected at 36 hpi and were assayed using TCID_50_. (**J and K**) Marc-145 cells were transfected with siNC or LC3 specific siRNA (50, 100 nM) for 12 h and then infected with PRRSV (MOI = 0.1). At 36 hpi, cells were collected, and the expression of PRRSV N protein was detected using western blotting; and for progeny viral titers analysis, supernatants were collected at 36 hpi and were analyzed using TCID_50_ assay. Data are mean ± SD values of three independent results. ^*^, *P* < 0.05; ^**^, *P* < 0.01; ^***^, *P* < 0.001; ns, not significant.

The next whether TRIM25 affected PRRSV replication *in vitro* was determined. First, the effect of PRRSV infection on TRIM25 expression was analyzed. Marc-145 cells infected with PRRSV or not were lysed 24, 36, and 48 hpi and analyzed by western blotting. The results showed that with the progression of viral infection, TRIM25 protein expression decreased gradually (Fig. 9A). Then Vector, Flag-TRIM25, shNC, and shTRIM25 cells were infected with PRRSV at an MOI of 0.1. Cells and supernatants were collected, respectively, at 24, 36, 48, 60, and 72 hpi, respectively, for analysis of viral protein expression by western blotting and viral replication kinetic feature by TCID_50_ assay. As shown in Fig. 9B, overexpression of TRIM25 markedly decreased the expression of PRRSV N protein at 24, 36, 48, 60, and 72 hpi, respectively. Meanwhile, compared with control group, overexpression of TRIM25 reduced supernatants progeny viral titers (decreased by 0.93, 0.83, 0.57, 0.72, and 0.54 lg, respectively) at all time points tested (Fig. 9C), indicating that overexpression of TRIM25 suppressed PRRSV replication *in vitro*. Similarly, knockdown of endogenous TRIM25 promoted the expression of N protein at all time points tested (Fig. 9D), and supernatants progeny viral titers increased by 0.89, 0.66, 0.79, 0.77, and 0.68 lg at 24, 36, 48, 60, and 72 hpi (Fig. 9E), respectively, indicating consistently slight enhancement of growth compared with the Vector control group.

Whether Nrf2 and p62 mediated the antiviral effect of TRIM25 was further investigated, both endogenous Nrf2 and p62 were knocked down using specific siRNA. Western blot results suggested that both of Nrf2 and p62 were effectively inhibited by specific siRNA (Fig. S10A and S11A). Knockdown of Nrf2 partially reversed the antiviral effect of TRIM25 (Fig. S10B and C), while knockdown of p62 completely reversed the antiviral effect of TRIM25 compared with control group (Fig. 9F and G), as indicated by the N protein level and supernatants viral titers (increased by 1.05 lg when the sip62 group compared with the siNC group) detection by western blot and TCID_50_ assay, indicating that TRIM25 exerts antiviral effect through the Nrf2-p62 pathway. Next, the impact of autophagy activity on PRRSV replication was elucidated. Marc-145 cells infected with PRRSV were treated with or without 3-MA. 3-MA treatment indeed blocked autophagy activity, as revealed by reduced LC3II/LC3I level. And 3-MA treatment dose-dependently decreased PRRSV N protein level when compared with the control group (Fig. 9H), and supernatants viral titers reduced by 1.43 and 2.06 lg, respectively, when compared with the control group (Fig. 9I). The effect of autophagy inhibition on the replication of PRRSV was further investigated by blocking autophagy pathway via knockdown the expression of LC3 using specific siRNA. The western blot results confirmed the knockdown efficiency of LC3 by specific siRNA (Fig. S11B). Silencing of LC3 concentration-dependently attenuated viral N protein level, and supernatant viral titers decreased by 1.17 and 1.59 lg, respectively, compared with the control group (Fig. 9J and K). Since it was demonstrated that TRIM25 inhibited KEAP-Nrf2-p62-mediated autophagy, these data suggest that TRIM25 inhibits PRRSV replication via inhibiting the KEAP1/Nrf2/p62-mediated autophagy.

## DISCUSSION

Ubiquitinated protein aggregates generated under various stress conditions such as oxidative stress, cytotoxic effects, and virus infection are generally adverse to the host. To eliminate adverse effects and restore protein metabolism balance, host cells activate the autophagy system through complex molecular mechanisms, which is one of the major cellular mechanisms contributing to protein homeostasis in the cell. In the present study, PRRSV infection reduced intracellular aggregates, and this phenotype was closely related to autophagy activation, as 3-MA treatment reversed the downward trend of aggregates caused by PRRSV (Fig. 1I and J). In fact, in addition to PRRSV, some other common animal-hosted viruses such as PEDV and PRV can also induce ER stress and autophagy, indicating that viruses are closely associated with aggregates formation and degradation. In the current study, TRIM25 inhibited the degradation of aggregates caused by PRRSV, and this was attributed to autophagy inhibition caused by TRIM25 since TRIM25 inhibited autophagy activation during PRRSV infection, suggesting that it acted as a negative regulator of autophagy. Previous research has shown that TRIM25 acted as a viral replication host restriction factor during PRRSV infection (42), and its inhibition of autophagy under normal physiological conditions effectively excluded its regulation on autophagy resulting from inhibition of PRRSV infection.

As an autophagy adaptor protein, p62 plays a dual role in autophagy. On the one hand, the intracellular p62 level is strictly regulated by autophagic activity, while, on the other hand, p62 can also negatively regulate the autophagic activity of cells by activating the mTORC1 signaling pathway. Therefore, it was speculated that the inhibition of autophagy activation by TRIM25 may be due to its inhibitory effect on p62 protein oligomerization, as the inhibition of autophagy caused by TRIM25 was accompanied by a significant increase in p62 protein expression (Fig. 3H and 4). p62 is regulated at multiple levels. At the transcriptional level, p62 expression is activated in a feedforward loop by Nrf2 (43). At the protein stability level, p62 is degraded along with protein aggregates by autophagy (38). At the post-translational level, p62 is phosphorylated at numerous sites, which enhances its activity (44–47). The present results showed that TRIM25 catalyzed the K63-linked ubiquitination of p62 and simultaneously suppressed its oligomerization (Fig. 4B through D). Previous research has demonstrated that the K63-linked ubiquitination of p62 suppresses its oligomerization (40). Therefore, it was speculated that TRIM25 may inhibit p62 oligomerization via promoting its K63-linked ubiquitination. In the present study, TRIM25 activated Nrf2, which is an activator of p62 (43), and the protein level of p62 markedly increased by TRIM25, indicating that TRIM25 upregulated p62 expression by activating the Nrf2 pathway.

Analysis of cell-insoluble fractions showed that, in addition to p62, TRIM25 was also a component of protein aggregates (Fig. 8), which further confirmed that TRIM25 was closely related to the formation of protein aggregates, and this association may be related to its interaction with key proteins in the aggregates; however, the exact molecular mechanism still needs further investigation. In fact, the accumulation of polyubiquitinated protein aggregates is a hallmark of several neurodegenerative disorders, as well as of a number of other protein aggregation diseases affecting muscles, heart, liver, and lung (48, 49). Previous research suggested that PRRSV Nsp2 was associated with protein aggresomes and could induce host cell aggrephagy (50). The present study showed that induction of protein aggregates may be a pathogenic mechanism of PRRSV, although the underlying mechanism remains unclear. TRIM25 negatively regulated PRRSV-induced autophagy activation (Fig. 6), and the present study revealed that TRIM25 suppressed PRRSV replication (Fig. 9), which was in agreement with previous research (42). Considering that TRIM25 substantially inhibited autophagy in the absence of PRRSV (Fig. 6), it was speculated that the inhibition of autophagy activation by TRIM25 was not attributed to the inhibition of PRRSV infection.

Few studies have systematically explored the association and mutual regulatory mechanisms between host cells and virus-induced aggregates. Therefore, the present study used PRRSV as a model virus to explore in detail its impact on the formation of protein aggregates in host cells and to explore how host cells regulate the formation and degradation of protein aggregates. The current results revealed that TRIM25 halted aggregates degradation through suppression of autophagy activation elicited during PRRSV infection. Thus, targeting TRIM25 and pathways involved in the regulation of protein aggregates may provide new insights for the development of novel therapeutic methods to reduce the pathogenicity of PRRSV.

## Data Availability

The data sets analyzed during the current study are available from the corresponding author on reasonable request.

## SUPPLEMENTAL MATERIAL

Supplemental material jvi.01437-24-s0001.docx Figures S1 to S11.

ASM does not own the copyrights to Supplemental Material that may be linked to, or accessed through, an article. The authors have granted ASM a non-exclusive, world-wide license to publish the Supplemental Material files. Please contact the corresponding author directly for reuse.

## References

[1] Kroemer G, Mariño G, Levine B. 2010. Autophagy and the integrated stress response. Mol Cell 40:280–293. doi:10.1016/j.molcel.2010.09.023 20965422 PMC3127250

[2] Balch WE, Morimoto RI, Dillin A, Kelly JW. 2008. Adapting proteostasis for disease intervention. Science 319:916–919. doi:10.1126/science.1141448 18276881

[3] Chin L-S, Olzmann JA, Li L. 2010. Parkin-mediated ubiquitin signalling in aggresome formation and autophagy. Biochem Soc Trans 38:144–149. doi:10.1042/BST0380144 20074049 PMC2846638

[4] Takalo M, Salminen A, Soininen H, Hiltunen M, Haapasalo A. 2013. Protein aggregation and degradation mechanisms in neurodegenerative diseases. Am J Neurodegener Dis 2:1–14. 23516262 PMC3601466

[5] Goldberg AL. 2003. Protein degradation and protection against misfolded or damaged proteins. Nature New Biol 426:895–899. doi:10.1038/nature02263 14685250

[6] Markossian KA, Kurganov BI. 2004. Protein folding, misfolding, and aggregation. Formation of inclusion bodies and aggresomes. Biochem (Moscow) 69:971–984. doi:10.1023/B:BIRY.0000043539.07961.4c 15521811

[7] Komatsu M, Kurokawa H, Waguri S, Taguchi K, Kobayashi A, Ichimura Y, Sou Y-S, Ueno I, Sakamoto A, Tong KI, Kim M, Nishito Y, Iemura S, Natsume T, Ueno T, Kominami E, Motohashi H, Tanaka K, Yamamoto M. 2010. The selective autophagy substrate p62 activates the stress responsive transcription factor Nrf2 through inactivation of Keap1. Nat Cell Biol 12:213–223. doi:10.1038/ncb2021 20173742

[8] Olanow CW, Perl DP, DeMartino GN, McNaught KSP. 2004. Lewy-body formation is an aggresome-related process: a hypothesis. Lancet Neurol 3:496–503. doi:10.1016/S1474-4422(04)00827-0 15261611

[9] Komatsu M, Waguri S, Chiba T, Murata S, Iwata J, Tanida I, Ueno T, Koike M, Uchiyama Y, Kominami E, Tanaka K. 2006. Loss of autophagy in the central nervous system causes neurodegeneration in mice. Nature New Biol 441:880–884. doi:10.1038/nature04723 16625205

[10] Johansen T, Lamark T. 2011. Selective autophagy mediated by autophagic adapter proteins. Autophagy 7:279–296. doi:10.4161/auto.7.3.14487 21189453 PMC3060413

[11] Kuusisto E, Salminen A, Alafuzoff I. 2001. Ubiquitin-binding protein p62 is present in neuronal and glial inclusions in human tauopathies and synucleinopathies. Neuroreport 12:2085–2090. doi:10.1097/00001756-200107200-00009 11447312

[12] Nagaoka U, Kim K, Jana NR, Doi H, Maruyama M, Mitsui K, Oyama F, Nukina N. 2004. Increased expression of p62 in expanded polyglutamine-expressing cells and its association with polyglutamine inclusions. J Neurochem 91:57–68. doi:10.1111/j.1471-4159.2004.02692.x 15379887

[13] Zatloukal K, Stumptner C, Fuchsbichler A, Heid H, Schnoelzer M, Kenner L, Kleinert R, Prinz M, Aguzzi A, Denk H. 2002. p62 Is a common component of cytoplasmic inclusions in protein aggregation diseases. Am J Pathol 160:255–263. doi:10.1016/S0002-9440(10)64369-6 11786419 PMC1867135

[14] Bjørkøy G, Lamark T, Brech A, Outzen H, Perander M, Overvatn A, Stenmark H, Johansen T. 2005. p62/SQSTM1 forms protein aggregates degraded by autophagy and has a protective effect on huntingtin-induced cell death. J Cell Biol 171:603–614. doi:10.1083/jcb.200507002 16286508 PMC2171557

[15] Katsuragi Y, Ichimura Y, Komatsu M. 2015. p62/SQSTM1 functions as a signaling hub and an autophagy adaptor. FEBS J 282:4672–4678. doi:10.1111/febs.13540 26432171

[16] Seibenhener ML, Babu JR, Geetha T, Wong HC, Krishna NR, Wooten MW. 2004. Sequestosome 1/p62 is a polyubiquitin chain binding protein involved in ubiquitin proteasome degradation. Mol Cell Biol 24:8055–8068. doi:10.1128/MCB.24.18.8055-8068.2004 15340068 PMC515032

[17] Itoh K, Chiba T, Takahashi S, Ishii T, Igarashi K, Katoh Y, Oyake T, Hayashi N, Satoh K, Hatayama I, Yamamoto M, Nabeshima Y. 1997. An Nrf2/small Maf heterodimer mediates the induction of phase II detoxifying enzyme genes through antioxidant response elements. Biochem Biophys Res Commun 236:313–322. doi:10.1006/bbrc.1997.6943 9240432

[18] Itoh K, Wakabayashi N, Katoh Y, Ishii T, Igarashi K, Engel JD, Yamamoto M. 1999. Keap1 represses nuclear activation of antioxidant responsive elements by Nrf2 through binding to the amino-terminal Neh2 domain. Genes Dev 13:76–86. doi:10.1101/gad.13.1.76 9887101 PMC316370

[19] Wakabayashi N, Itoh K, Wakabayashi J, Motohashi H, Noda S, Takahashi S, Imakado S, Kotsuji T, Otsuka F, Roop DR, Harada T, Engel JD, Yamamoto M. 2003. Keap1-null mutation leads to postnatal lethality due to constitutive Nrf2 activation. Nat Genet 35:238–245. doi:10.1038/ng1248 14517554

[20] Kobayashi A, Kang M-I, Okawa H, Ohtsuji M, Zenke Y, Chiba T, Igarashi K, Yamamoto M. 2004. Oxidative stress sensor Keap1 functions as an adaptor for Cul3-based E3 ligase to regulate proteasomal degradation of Nrf2. Mol Cell Biol 24:7130–7139. doi:10.1128/MCB.24.16.7130-7139.2004 15282312 PMC479737

[21] Ishii T, Itoh K, Takahashi S, Sato H, Yanagawa T, Katoh Y, Bannai S, Yamamoto M. 2000. Transcription factor Nrf2 coordinately regulates a group of oxidative stress-inducible genes in macrophages. J Biol Chem 275:16023–16029. doi:10.1074/jbc.275.21.16023 10821856

[22] Mandell MA, Jain A, Arko-Mensah J, Chauhan S, Kimura T, Dinkins C, Silvestri G, Münch J, Kirchhoff F, Simonsen A, Wei Y, Levine B, Johansen T, Deretic V. 2014. TRIM proteins regulate autophagy and can target autophagic substrates by direct recognition. Dev Cell 30:394–409. doi:10.1016/j.devcel.2014.06.013 25127057 PMC4146662

[23] Mandell MA, Kimura T, Jain A, Johansen T, Deretic V. 2014. TRIM proteins regulate autophagy: TRIM5 is a selective autophagy receptor mediating HIV-1 restriction. Autophagy 10:2387–2388. doi:10.4161/15548627.2014.984278 25587751 PMC4502693

[24] Kimura T, Jain A, Choi SW, Mandell MA, Schroder K, Johansen T, Deretic V. 2015. TRIM-mediated precision autophagy targets cytoplasmic regulators of innate immunity. J Cell Biol 210:973–989. doi:10.1083/jcb.201503023 26347139 PMC4576868

[25] Jena KK, Kolapalli SP, Mehto S, Nath P, Das B, Sahoo PK, Ahad A, Syed GH, Raghav SK, Senapati S, Chauhan S, Chauhan S. 2018. TRIM16 controls assembly and degradation of protein aggregates by modulating the p62-NRF2 axis and autophagy. EMBO J 37:e98358. doi:10.15252/embj.201798358 30143514 PMC6138442

[26] Lyu L, Chen Z, McCarty N. 2022. TRIM44 links the UPS to SQSTM1/p62-dependent aggrephagy and removing misfolded proteins. Autophagy 18:783–798. doi:10.1080/15548627.2021.1956105 34382902 PMC9037492

[27] Chang J, Hwang HJ, Kim B, Choi Y-G, Park J, Park Y, Lee BS, Park H, Yoon MJ, Woo J-S, Kim C, Park M-S, Lee J-B, Kim YK. 2021. TRIM28 functions as a negative regulator of aggresome formation. Autophagy 17:4231–4248. doi:10.1080/15548627.2021.1909835 33783327 PMC8726693

[28] Liu Y, Tao S, Liao L, Li Y, Li H, Li Z, Lin L, Wan X, Yang X, Chen L. 2020. TRIM25 promotes the cell survival and growth of hepatocellular carcinoma through targeting Keap1-Nrf2 pathway. Nat Commun 11:348. doi:10.1038/s41467-019-14190-2 31953436 PMC6969153

[29] Cavanagh D. 1997. Nidovirales: a new order comprising Coronaviridae and Arteriviridae. Arch Virol 142:629–633. 9349308

[30] Wensvoort G, Terpstra C, Pol JMA, ter Laak EA, Bloemraad M, de Kluyver EP, Kragten C, van Buiten L, den Besten A, Wagenaar F, Broekhuijsen JM, Moonen PLJM, Zetstra T, de Boer EA, Tibben HJ, de Jong MF, van ‘t Veld P, Greenland GJR, van Gennep JA, Voets MTh, Verheijden JHM, Braamskamp J. 1991. Mystery swine disease in the Netherlands: the isolation of Lelystad virus. Vet Q 13:121–130. doi:10.1080/01652176.1991.9694296 1835211

[31] Gao P, Chai Y, Song J, Liu T, Chen P, Zhou L, Ge X, Guo X, Han J, Yang H. 2019. Reprogramming the unfolded protein response for replication by porcine reproductive and respiratory syndrome virus. PLoS Pathog 15:e1008169. doi:10.1371/journal.ppat.1008169 31738790 PMC6932825

[32] Zou D, Xu J, Duan X, Xu X, Li P, Cheng L, Zheng L, Li X, Zhang Y, Wang X, Wu X, Shen Y, Yao X, Wei J, Yao L, Li L, Song B, Ma J, Liu X, Wu Z, Zhang H, Cao H. 2019. Porcine epidemic diarrhea virus ORF3 protein causes endoplasmic reticulum stress to facilitate autophagy. Vet Microbiol 235:209–219. doi:10.1016/j.vetmic.2019.07.005 31383304 PMC7117398

[33] Yang S, Zhu J, Zhou X, Wang H, Li X, Zhao A. 2019. Induction of the unfolded protein response (UPR) during pseudorabies virus infection. Vet Microbiol 239:108485. doi:10.1016/j.vetmic.2019.108485 31767094

[34] Zhang A, Duan H, Zhao H, Liao H, Du Y, Li L, Jiang D, Wan B, Wu Y, Ji P, Zhou E-M, Zhang G. 2020. Interferon-induced transmembrane protein 3 is a virus-associated protein which suppresses porcine reproductive and respiratory syndrome virus replication by blocking viral membrane fusion. J Virol 94:e01350-20. doi:10.1128/JVI.01350-20 PMC7925183 32999030

[35] Fusco C, Micale L, Egorov M, Monti M, D’Addetta EV, Augello B, Cozzolino F, Calcagnì A, Fontana A, Polishchuk RS, Didelot G, Reymond A, Pucci P, Merla G. 2012. The E3-ubiquitin ligase TRIM50 interacts with HDAC6 and p62, and promotes the sequestration and clearance of ubiquitinated proteins into the aggresome. PLoS One 7:e40440. doi:10.1371/journal.pone.0040440 22792322 PMC3392214

[36] Sun M-X, Huang L, Wang R, Yu Y-L, Li C, Li P-P, Hu X-C, Hao H-P, Ishag HA, Mao X. 2012. Porcine reproductive and respiratory syndrome virus induces autophagy to promote virus replication. Autophagy 8:1434–1447. doi:10.4161/auto.21159 22739997

[37] Itoh K, Wakabayashi N, Katoh Y, Ishii T, O’Connor T, Yamamoto M. 2003. Keap1 regulates both cytoplasmic-nuclear shuttling and degradation of Nrf2 in response to electrophiles. Genes Cells 8:379–391. doi:10.1046/j.1365-2443.2003.00640.x 12653965

[38] Pankiv S, Clausen TH, Lamark T, Brech A, Bruun J-A, Outzen H, Øvervatn A, Bjørkøy G, Johansen T. 2007. p62/SQSTM1 binds directly to Atg8/LC3 to facilitate degradation of ubiquitinated protein aggregates by autophagy. J Biol Chem 282:24131–24145. doi:10.1074/jbc.M702824200 17580304

[39] Moscat J, Diaz-Meco MT. 2009. p62 at the crossroads of autophagy, apoptosis, and cancer. Cell 137:1001–1004. doi:10.1016/j.cell.2009.05.023 19524504 PMC3971861

[40] Pan J-A, Sun Y, Jiang Y-P, Bott AJ, Jaber N, Dou Z, Yang B, Chen J-S, Catanzaro JM, Du C, Ding W-X, Diaz-Meco MT, Moscat J, Ozato K, Lin RZ, Zong W-X. 2016. TRIM21 ubiquitylates SQSTM1/p62 and suppresses protein sequestration to regulate redox homeostasis. Mol Cell 62:149–151. doi:10.1016/j.molcel.2016.03.015 27058791

[41] Komatsu M, Waguri S, Koike M, Sou Y-S, Ueno T, Hara T, Mizushima N, Iwata J-I, Ezaki J, Murata S, Hamazaki J, Nishito Y, Iemura S-I, Natsume T, Yanagawa T, Uwayama J, Warabi E, Yoshida H, Ishii T, Kobayashi A, Yamamoto M, Yue Z, Uchiyama Y, Kominami E, Tanaka K. 2007. Homeostatic levels of p62 control cytoplasmic inclusion body formation in autophagy-deficient mice. Cell 131:1149–1163. doi:10.1016/j.cell.2007.10.035 18083104

[42] Zhao K, Li L-W, Jiang Y-F, Gao F, Zhang Y-J, Zhao W-Y, Li G-X, Yu L-X, Zhou Y-J, Tong G-Z. 2019. Nucleocapsid protein of porcine reproductive and respiratory syndrome virus antagonizes the antiviral activity of TRIM25 by interfering with TRIM25-mediated RIG-I ubiquitination. Vet Microbiol 233:140–146. doi:10.1016/j.vetmic.2019.05.003 31176400 PMC7117424

[43] Jain A, Lamark T, Sjøttem E, Larsen KB, Awuh JA, Øvervatn A, McMahon M, Hayes JD, Johansen T. 2010. p62/SQSTM1 is a target gene for transcription factor NRF2 and creates a positive feedback loop by inducing antioxidant response element-driven gene transcription. J Biol Chem 285:22576–22591. doi:10.1074/jbc.M110.118976 20452972 PMC2903417

[44] Ichimura Y, Waguri S, Sou Y-S, Kageyama S, Hasegawa J, Ishimura R, Saito T, Yang Y, Kouno T, Fukutomi T, Hoshii T, Hirao A, Takagi K, Mizushima T, Motohashi H, Lee M-S, Yoshimori T, Tanaka K, Yamamoto M, Komatsu M. 2013. Phosphorylation of p62 activates the Keap1-Nrf2 pathway during selective autophagy. Mol Cell 51:618–631. doi:10.1016/j.molcel.2013.08.003 24011591

[45] Lim J, Lachenmayer ML, Wu S, Liu W, Kundu M, Wang R, Komatsu M, Oh YJ, Zhao Y, Yue Z. 2015. Proteotoxic stress induces phosphorylation of p62/SQSTM1 by ULK1 to regulate selective autophagic clearance of protein aggregates. PLoS Genet 11:e1004987. doi:10.1371/journal.pgen.1004987 25723488 PMC4344198

[46] Linares JF, Amanchy R, Greis K, Diaz-Meco MT, Moscat J. 2011. Phosphorylation of p62 by cdk1 controls the timely transit of cells through mitosis and tumor cell proliferation. Mol Cell Biol 31:105–117. doi:10.1128/MCB.00620-10 20974803 PMC3019844

[47] Tanji K, Miki Y, Ozaki T, Maruyama A, Yoshida H, Mimura J, Matsumiya T, Mori F, Imaizumi T, Itoh K, Kakita A, Takahashi H, Wakabayashi K. 2014. Phosphorylation of serine 349 of p62 in Alzheimer’s disease brain. Acta Neuropathol Commun 2:50. doi:10.1186/2051-5960-2-50 24886973 PMC4035093

[48] Levine B, Kroemer G. 2008. Autophagy in the pathogenesis of disease. Cell 132:27–42. doi:10.1016/j.cell.2007.12.018 18191218 PMC2696814

[49] Luciani A, Villella VR, Esposito S, Brunetti-Pierri N, Medina D, Settembre C, Gavina M, Pulze L, Giardino I, Pettoello-Mantovani M, D’Apolito M, Guido S, Masliah E, Spencer B, Quaratino S, Raia V, Ballabio A, Maiuri L. 2010. Defective CFTR induces aggresome formation and lung inflammation in cystic fibrosis through ROS-mediated autophagy inhibition. Nat Cell Biol 12:863–875. doi:10.1038/ncb2090 20711182

[50] Cao S, Liu J, Ding G, Shao Q, Wang B, Li Y, Feng J, Zhao Y, Liu S, Xiao Y. 2020. The tail domain of PRRSV NSP2 plays a key role in aggrephagy by interacting with 14-3-3ε. Vet Res 51:104. doi:10.1186/s13567-020-00816-7 32811532 PMC7433210

