# Supplementary material for: Tripartite motif 25 inhibits protein aggregate degradation during PRRSV infection by suppressing p62-mediated autophagy
Source: J Virol. 2024 Oct 31;98(11):e01437-24. doi: 10.1128/jvi.01437-24 (PMC11575163; doi:10.1128/jvi.01437-24)
Supplement: Supplemental material — Figures S1 to S11. [file jvi.01437-24-s0001.docx]

**Supplementary Materials and Methods**

**
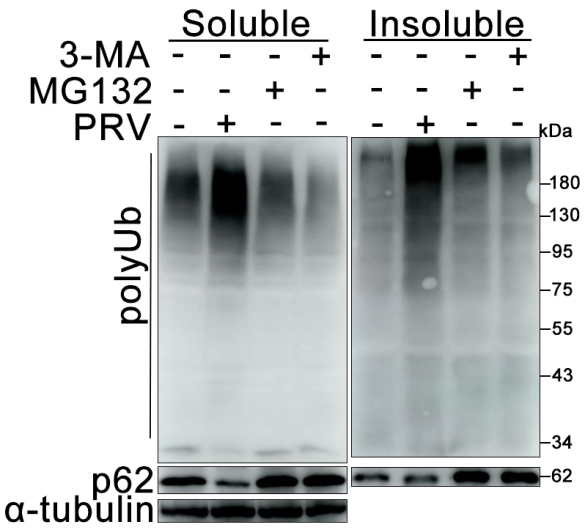
**

**FIG S1 PRV infection promotes ubiquitinated protein aggregates accumulation in PK-15 cells.** Effect of PRV infection on ubiquitinated protein aggregates formation. PK-15 cells were infected or not with PRV (MOI=0.1). At 36 hpi, cells were harvested and lysated using NP40 lysis buffer. After centrifugation, the clear supernatants were used as soluble fraction. Cell debris was dissolved in 8 M urea and was used as insoluble fraction.


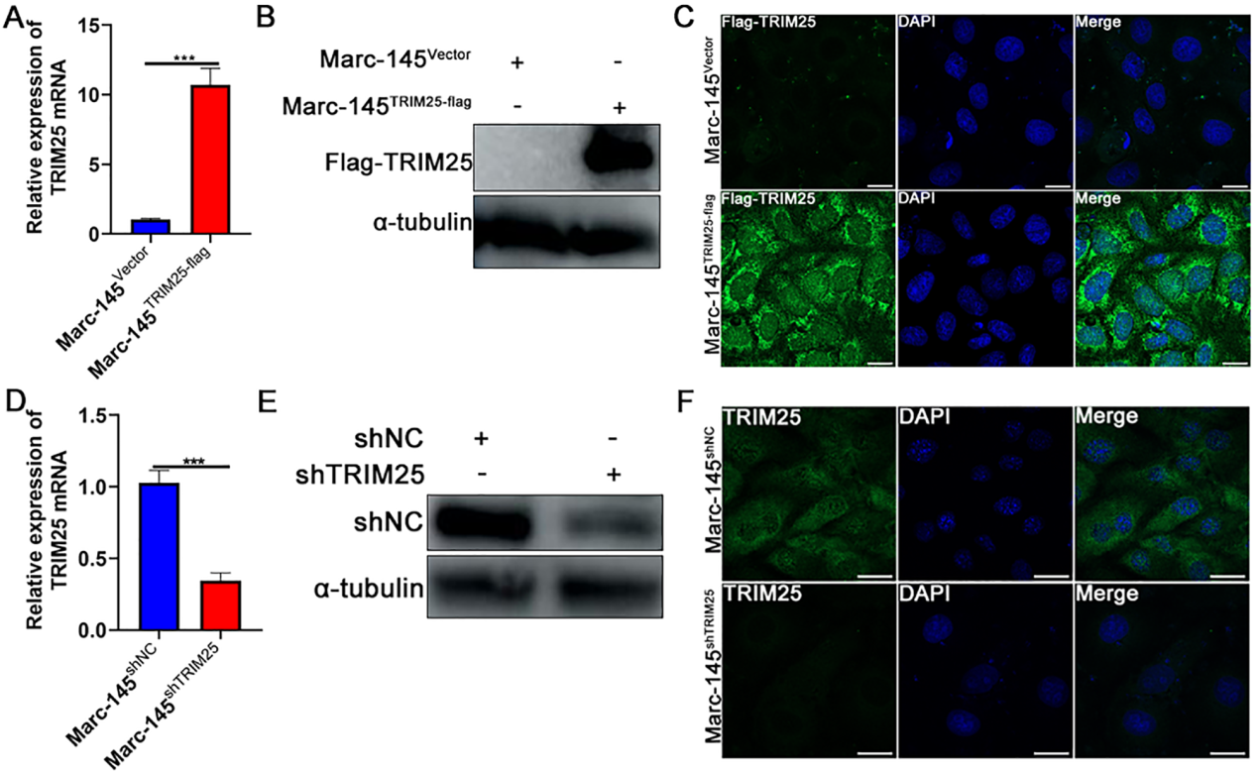


**FIG S2 Identification of recombinant Marc-145 cell lines with TRIM25 overexpression or knockdown.** pTRIP-TRIM25-flag expression vector (1.3 μg) was cotransfected into HEK293T cells, with packaging vectors psPAX2 (1.8 μg) and pMD2.G (1.0 μg) to produce pseudotyped lentiviral vectors using X-tremeGENE HP DNA transfection reagent. Supernatants containing the pseudotyped lentiviruses were collected 48 hours post transfection (hpt), followed by centrifuging at 1,000 rpm for 5 min at 4 ˚C to remove cell debris. Marc-145 cells were infected with pseudovirions in the presence of 1 μg/ml polybrene. A total of 48 h after infection, cells were selected in 10% FBS + Dulbecco modified Eagle medium (DMEM) containing 1 mg/ml puromycin. After selection for 2 weeks, cells were collected and identified using qPCR (A), Western blotting (B), and indirect fluorescence assay (IFA) (C), respectively. (D-F) To generate Marc-145 cell lines with TRIM25 knockdown, Marc-145 cells were infected with recombinant pseudovirions and selected in 10% FBS + DMEM containing 1 mg/ml puromycin. For knockdown cell line, lentiviral particles were generated via transfection of HEK293T cells with psPAX2, pMD2.G, and pLKO.1-puro constructs expressing short hairpin RNA (shRNA) against TRIM25 or empty vector control expressing a stuffer sequence. After selecting for 2 weeks using puromycin, cells were harvested and identified using qPCR (D), Western blotting (E), and IFA (F), respectively. ^***^, *P* < 0.001. Scale bar: 100 μm.


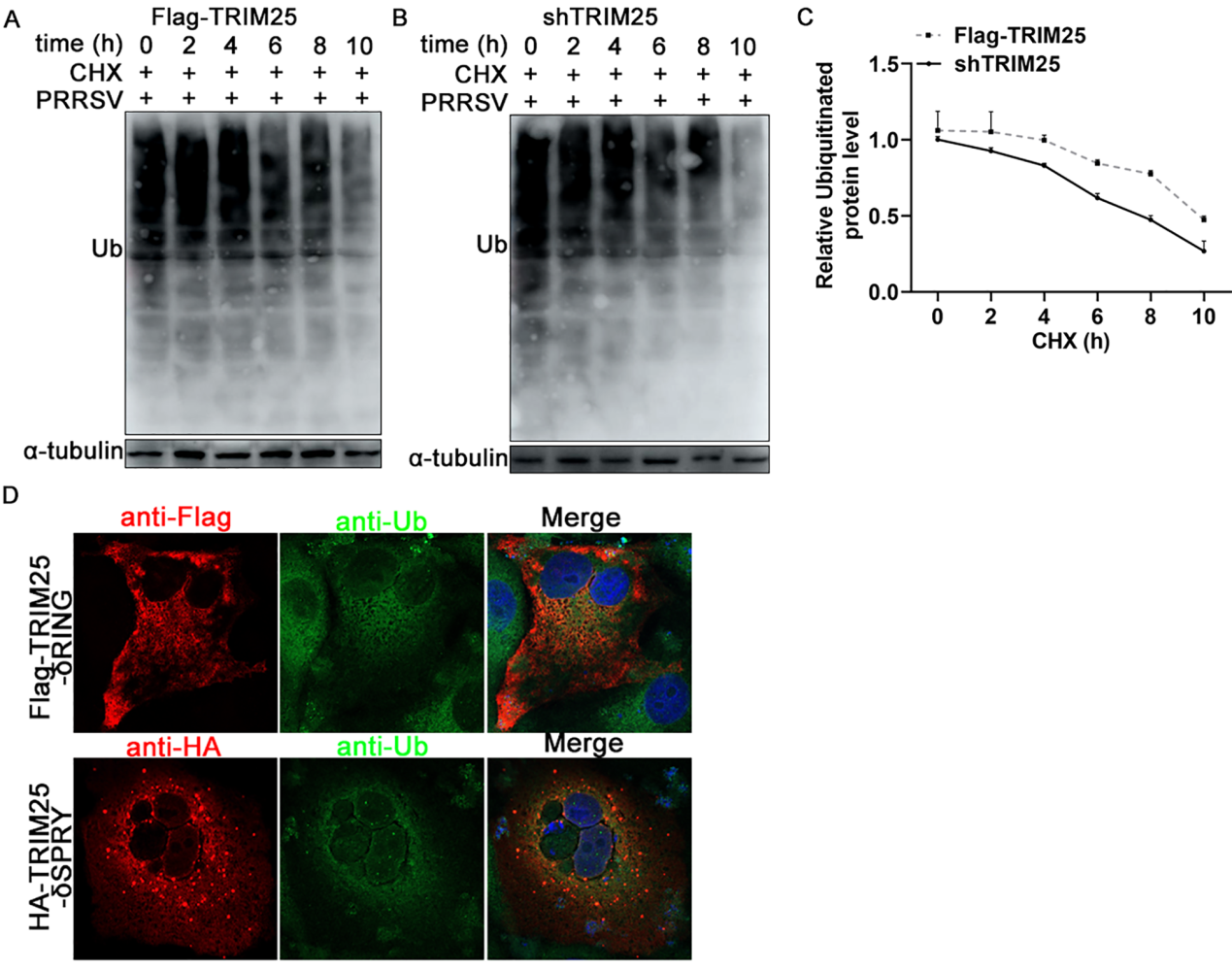


**FIG S3 TRIM25 destabilized intracellular ubiquitin via its E3 ligase activity.** (A and B) Flag-TRIM25 and shTRIM25 cells were infected with 0.1 MOI of PRRSV. At 12 hpi, cells were treated with 100 mg/ml of cycloheximide for 0, 2, 4, 6, 8, 10 h, then cells were harvested at indicated time points for analysis of intracellular ubiquitin level using Western blotting, and ubiquitin was normalized to α-tubulin and quantified with BandScan software (C). (D) Marc-145 cells seeded in 24-well plates were transfected with 500 ng of Flag-TRIM25-ΔRING or -ΔSPRY mutant plasmids. At 36 hpt, cells were fixed, permeabilized, and stained with mouse anti-flag monoclonal antibody (mAb), -HA mAb, or rabbit anti-ubiquitin polyclonal antibody, followed by incubating with Alexa 594-conjugated goat anti-mouse IgG (H+L) (red) and Alexa 488-conjugated goat anti-rabbit IgG (H+L) (green). Nuclei were stained using DAPI. Scale bar: 10 μm.

**
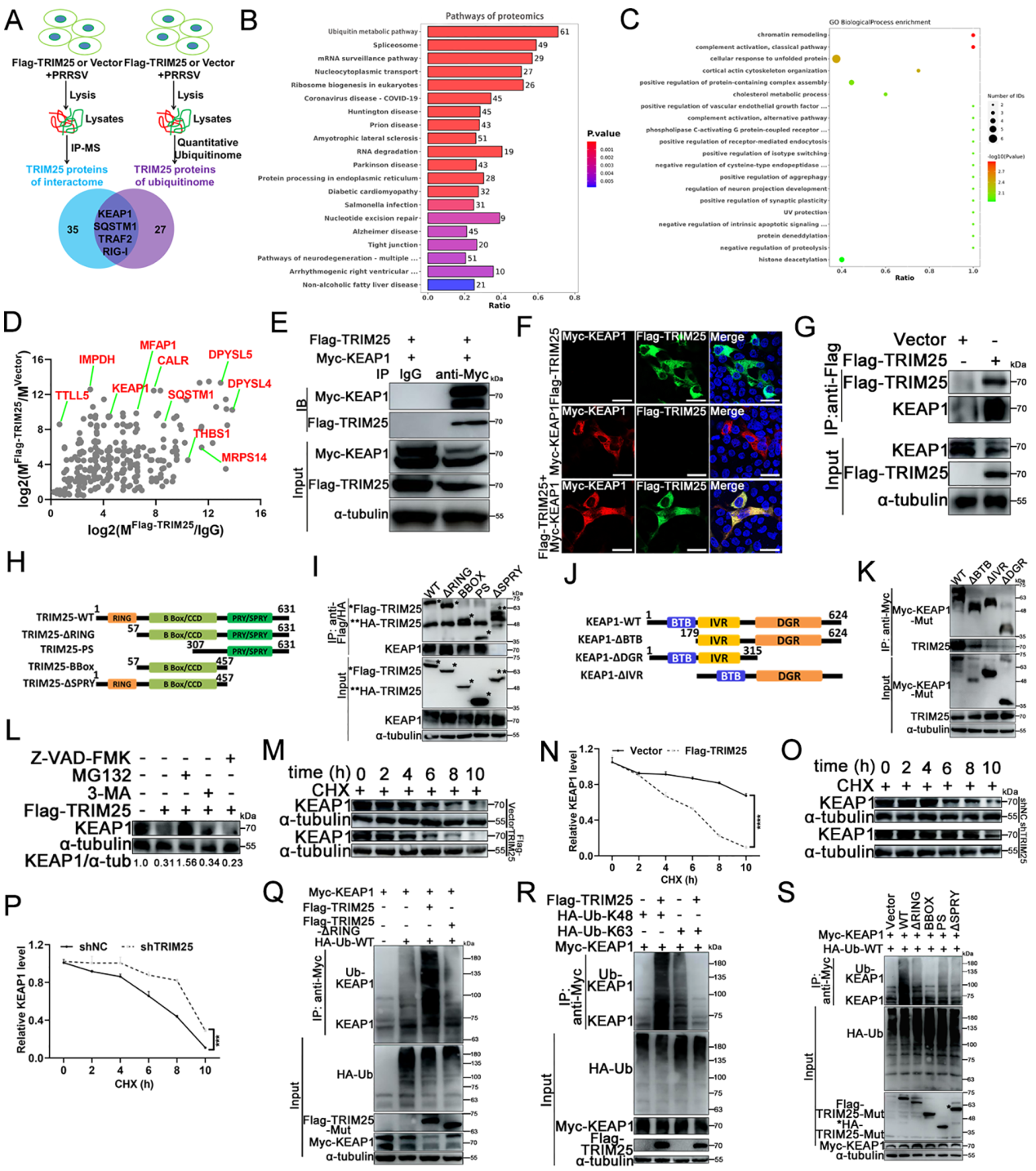
**

**FIG S4 TRIM25 catalyzes KEAP1 degradation via the ubiquitin-proteasome pathway.** (A) Scheme showing the procedure of identifying the downstream targets of TRIM25 by the intersection analysis of IP-MS and quantitative ubiquitinomics. (B) KEGG analysis of the enrichment of TRIM25-associated signaling pathway. (C) GO analysis of the enrichment of TRIM25-associated biological processes. (D) A volcano plot showing DEGs (red, upregulated genes; blue, downregulated genes) in Marc-145 cells overexpression of TRIM25. (E and F) Co-IP and confocal analysis of exogenous TRIM25-KEAP1 interaction in HEK293T cells. Scale bars: 10 μm. (G) Co-IP analysis of exogenous TRIM25 interaction with endogenous KEAP1 in Marc-145 cells. (H) The RING zinc-finger, B-Box, coiled-coil (CC) and PRY/SPRY (PS) domains of TRIM25 were indicated. (I) Co-IP identification of the key domain(s) in TRIM25 interacting with KEAP1. (J) Schematic diagram of KEAP1 truncated mutants, and the BTB, IVR and DGR domains were indicated. (K) Co-IP analysis the key domain(s) determining the interaction between KEAP1 and TRIM25. (L) Flag-TRIM25 cells were treated with MG132 (10 μM), 3-MA (10 mM) or Z-VAD-FMK (50 μM) for 12 h, then the expression of KEAP1 was analyzed. (M-P) Vector, Flag-TRIM25, shNC and shTRIM25 cells were treated with 100 mg/ml of CHX for 0, 2, 4, 6, 8, 10 h, respectively, cells were harvested for analysis of KEAP1 expression using Western blotting, and KEAP1 was normalized to α-tubulin and quantified with BandScan software. (Q and S) Co-IP analysis of the ubiquitination of KEAP1 catalyzed by TRIM25 (Q), or by its specific domain(s) (S). (R) Co-IP analysis of K48- or K63-linked ubiquitination of KEAP1 catalyzed by TRIM25.


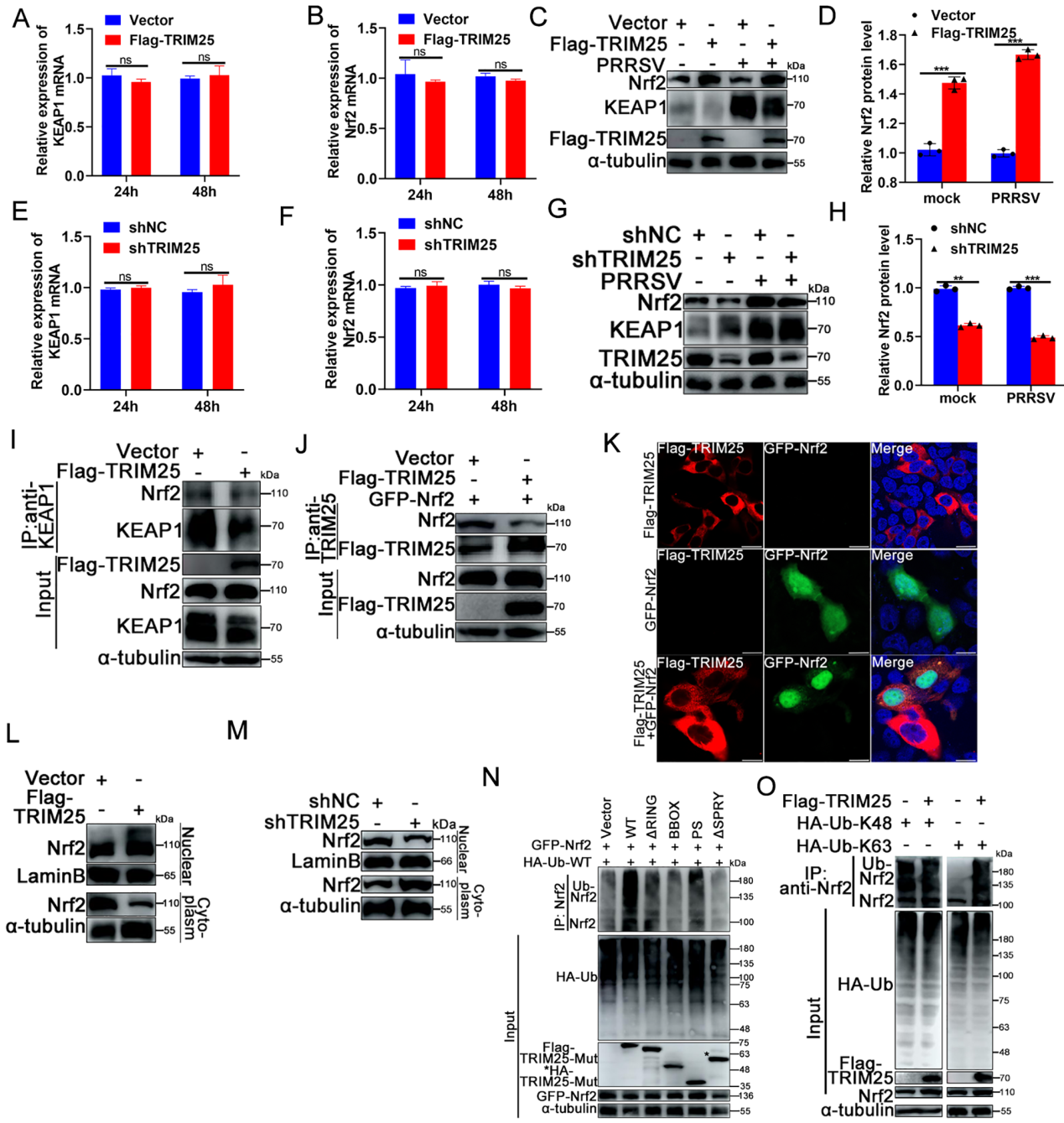


**FIG S5 TRIM25 activates Nrf2-mediated pathway via degradation of KEAP1.**

(A-D) Effect of TRIM25 overexpression on the expression of KEAP1 and Nrf2 mRNA or protein level in the presence of PRRSV or not. (E-H) Effect of TRIM25 knockdown on the expression of KEAP1 and Nrf2 mRNA or protein expression in the presence of PRRSV or not. (I) Effect of TRIM25 overexpression on KEAP1-Nrf2 interaction. Vector and Flag-TRIM25 plasmids were transfected into HEK293T cells for 48 h, cells were collected, lysated and then IP was performed with anti-KEAP1 mAb. IP products were detected using anti-KEAP1, -Nrf2 mAb. (J) Effect of TRIM25 overexpression on TRIM25-Nrf2 interaction. Flag-TRIM25 and GFP-Nrf2 plasmids were co-transfected into HEK293T cells for 48 h. Cells were harvested for co-IP analysis and the IP products were detected with anti-Nrf2 and -flag mAb. (K) Effect of TRIM25 on Nrf2 subcellular distribution. GFP-Nrf2 plasmid was co-transfected or not with Flag-TRIM25 plasmid into HEK293T cells. At 36 hpt, cells were fixed, permeabilized and stained with anti-flag mAb. Scale bar: 10 μm. (L and M) Nuclear/cytosolic fraction assay and Western blotting analysis of Nrf2 distribution. (N) Co-IP analysis of whether TRIM25 affects Nrf2 ubiquitination and the key domains of E3 ligase activity. (O) Co-IP analysis of K48- or K63-linked ubiquitination of Nrf2 by TRIM25. Data are mean ± SD values of three independent results. ^*^, *P* < 0.05; ^**^, *P* < 0.01; ns, not significant.


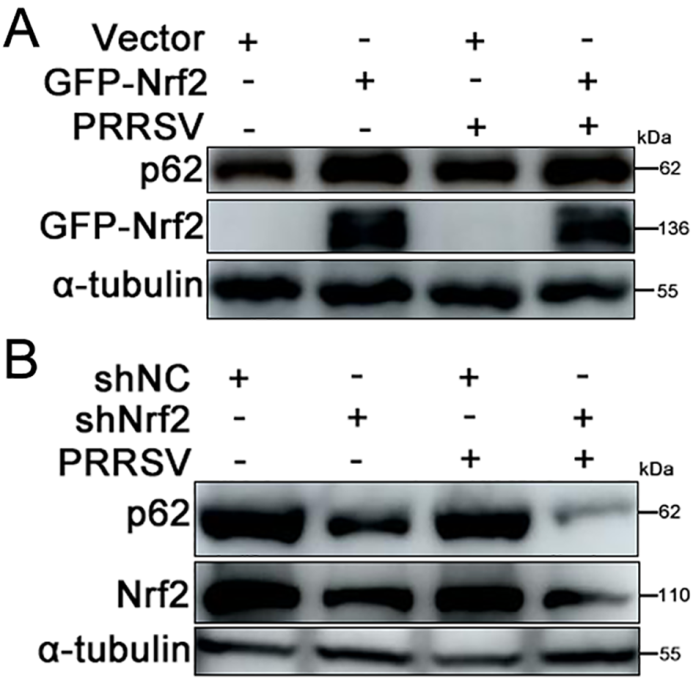


**FIG S6** (A) Effect of Nrf2 overexpression on p62 expression in the presence or absence of PRRSV. Vector or Flag-TRIM25 cell lines were plated into 6-well plates. After culturing for 24 h, cells were infected with 0.1 MOI of PRRSV or not. At 36 hpi, cells were harvested to detect the expression of p62 and GFP-Nrf2. (B) Effect of Nrf2 knockdown on p62 expression in the presence or absence of PRRSV. shNC or shTRIM25 cell lines in 6-well plates were infected with 0.1 MOI of PRRSV or not. At 36 hpi, cells were harvested to detect the expression of p62 and GFP-Nrf2.


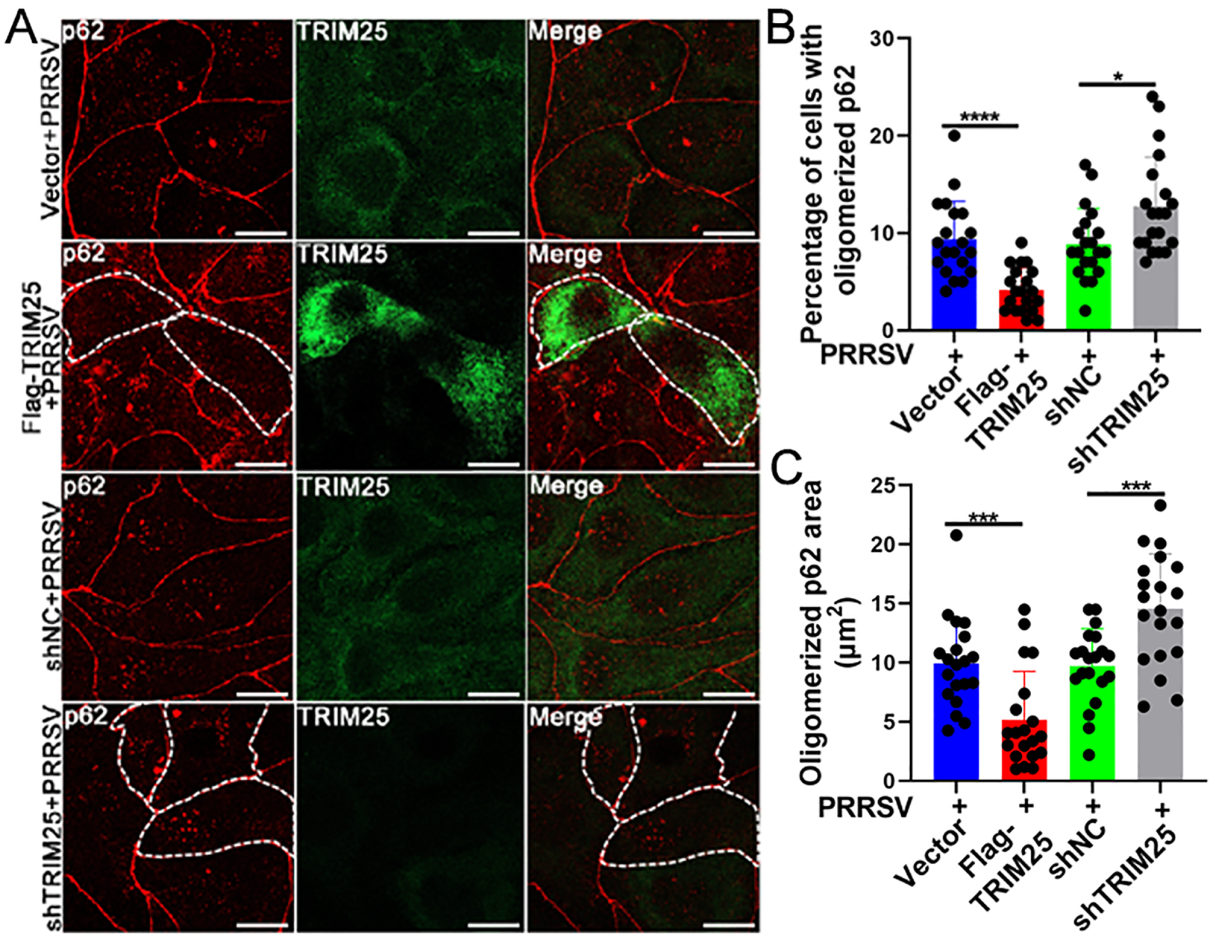


**FIG S7 Effect of TRIM25 on p62 oligomerization.** Vector, Flag-TRIM25, shNC and shTRIM25 cells infected with PRRSV (0.1 MOI) were stained with anti-flag and -p62 mAb and corresponding fluorescent second antibody. Oligomerized p62 puncta in the cells were statistically analyzed. The cells inside the dashed box were compared with the surrounding cells. Three independent experiments were performed, 20 independent fields of cells were calculated each time and typical data was shown. ^*^, *P* < 0.05; ^***^, *P* < 0.001; ^***^, *P* < 0.0001. Scale bar: 10 μm.


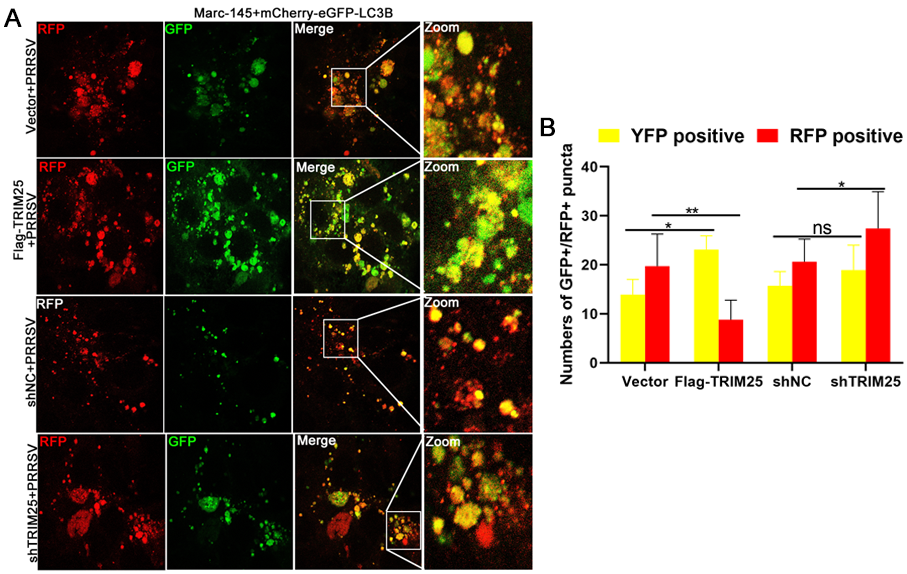


**FIG S8 TRIM25 inhibits PRRSV-induced autophagy activation.** (A) Vector, Flag-TRIM25, shNC and shTRIM25 cells were transfected with GFP-RFP-LC3B, and followed by infecting with PRRSV (0.1MOI). At 36 hpi, cells were fixed and assayed for the appearance of autophagy flux by confocal microscopy. Scale bars: 10 μm. (B) Graphic presentation of the average number of fluorescent structures present per cell from Fig. S8A. The number of fluorescent bodies per cell in 20 cells was counted for each data point shown (each bar). ^*^, *P* < 0.05; ^**^, *P* < 0.01; ns, no significant.


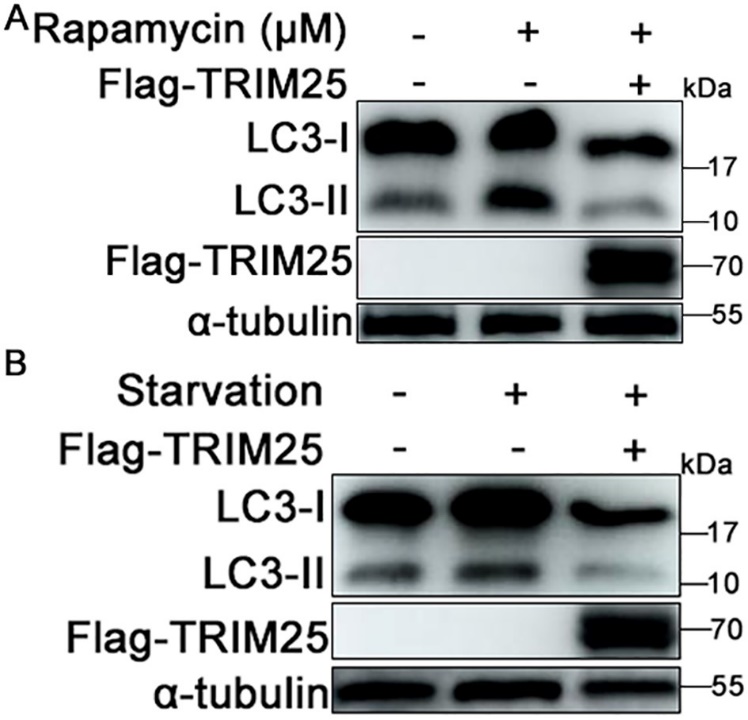


**FIG S9 TRIM25 suppresses starvation- and rapamycin-induced autophagy activation.** (A) Vector and Flag-TRIM25 cells were starved or not for 24 h and then cells were harvested to analyze the expression of LC3 protein. (B) Vector and Flag-TRIM25 cells were treated or not with 1 μM rapamycin for 24 h and then cells were subjected to western blotting analysis of LC3 protein expression.


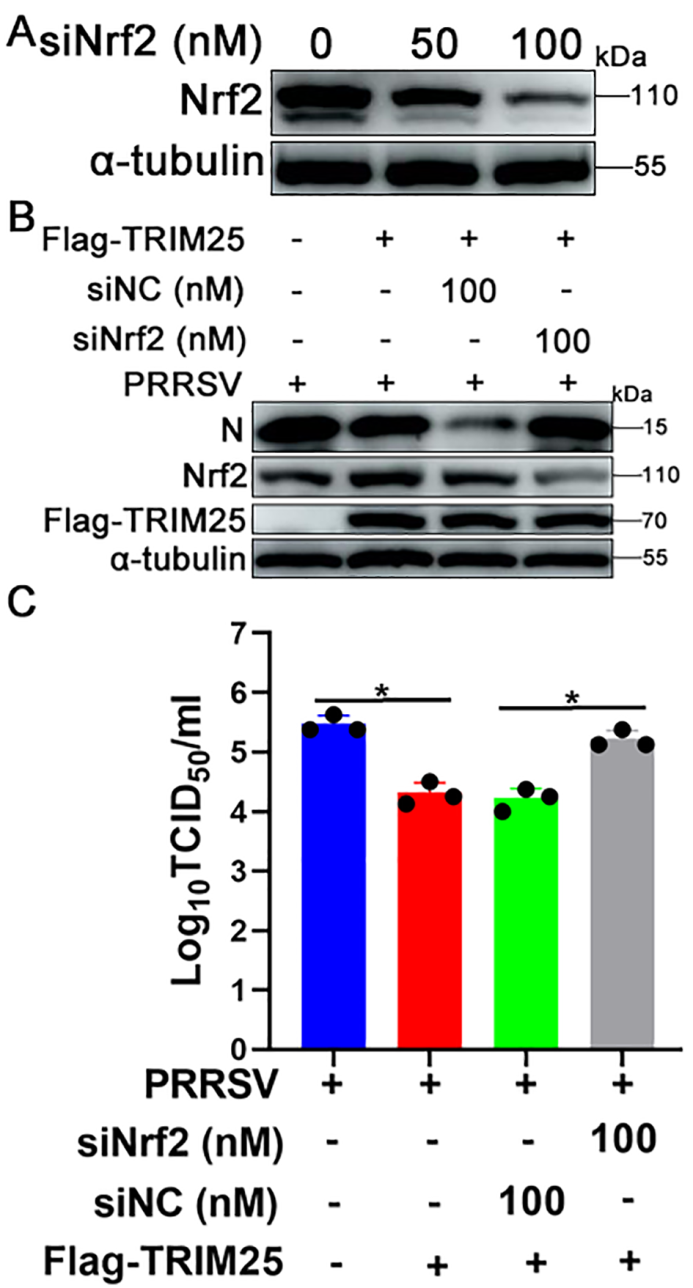


**FIG S10 Nrf2 mediates the anti-PRRSV activity of TRIM25.** (A) Marc-145 cells were transfected with 100 nM siNC or 50, 100 nM specific siRNA targeting Nrf2. At 36 hpt, cells were collected for detection of Nrf2 using western blotting. (B and C) TRIM25-Flag cells were transfected with 100 nM of siNC or siNrf2 for 12 h and then infected with 0.1 MOI of PRRSV. Cells were collected at 36 hpi to determine the expression of RRSV N protein using western blotting (A), supernatants were collected at 36 hpi to detect supernatants viral titers using TCID_50_ assay (B). Data are mean ± SD values of three independent results. ^***^, *P* < 0.001.


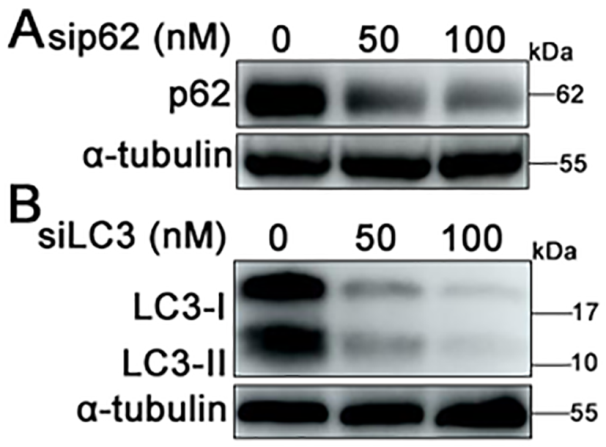


**FIG S11 Knockdown efficiency of specific siRNA targeting p62 and LC3.** Marc-145 cells were transfected with 100 nM siNC or 50, 100 nM specific siRNA targeting p62 or LC3, respectively. At 36 hpt, cells were collected for detection of p62 (A) and LC3 (B) using western blotting.
